# A three-groups non-local model for combining heterogeneous data sources to identify genes associated with Parkinson’s disease

**DOI:** 10.1093/biomtc/ujag090

**Published:** 2026-05-30

**Authors:** Troy P Wixson, Benjamin A Shaby, Daisy L Philtron, Leandro A Lima, Stacia K Wyman, Julia A Kaye, Steven Finkbeiner

**Affiliations:** Department of Statistics, Colorado State University, Fort Collins, CO 80523, United States; Department of Statistics, Colorado State University, Fort Collins, CO 80523, United States; Department of Applied Mathematics and Statistics, Colorado School of Mines, Golden, CO 80401, United States; Center for Systems and Therapeutics, Gladstone Institutes, San Francisco, CA 94158, United States; Innovative Genomics Institute, University of California Berkeley, Berkeley, CA 94720, United States; Center for Systems and Therapeutics, Gladstone Institutes, San Francisco, CA 94158, United States; Center for Systems and Therapeutics, Gladstone Institutes, San Francisco, CA 94158, United States; Taube/Koret Center for Neurodegenerative Disease, Gladstone Institutes, San Francisco, CA 94158, United States; Department of Neurology and Physiology, University of California San Francisco, San Francisco, CA 94143, United States

**Keywords:** GWAS, non-local prior, RNA-seq, variable selection

## Abstract

We seek to identify genes involved in Parkinson’s disease (PD) by combining information across different experiment types. Each experiment, taken individually, may contain too little information to distinguish some important genes from incidental ones. However, when experiments are combined using the proposed statistical framework, additional power emerges. The fundamental building block of the family of statistical models that we propose is a hierarchical three-groups mixture of distributions. Each gene is modeled probabilistically as belonging to either a null group that is unassociated with PD, a deleterious group, or a beneficial group. This three-groups formalism has two key features. By apportioning prior probability of group assignments with a Dirichlet distribution, the resultant posterior group probabilities automatically account for the multiplicity inherent in analyzing many genes simultaneously. By building models for experimental outcomes conditionally on the group labels, any number of data modalities may be combined in a single coherent probability model, allowing information sharing across experiment types. These two features result in parsimonious inference with few false positives, while simultaneously enhancing power to detect signals. Simulations show that our three-groups approach performs at least as well as commonly-used tools for GWAS and RNA-seq, and in some cases it performs better. We apply our proposed approach to publicly available genome-wide association studies and RNA-seq datasets, discovering novel genes that are potential therapeutic targets.

## 1 Introduction

A substantial portion of the risk of developing even so-called “sporadic disease” is attributable to genetic variants harbored by an individual. For this reason, huge genetic studies have investigated many major human diseases to uncover genetic variants that either directly cause or modify disease, first with the analysis of single-nucleotide variants (SNVs), then whole exomes, and now, increasingly, whole genomes. Conventionally, the analysis of genomic data focused on genome-wide association studies (GWAS), which have been used to identify associations between genetic variants and disease incidence or progression (Uffelmann et al., 2021).

However, the variants identified from GWAS of complex traits, such as human disease, usually explain only a small fraction of the known heritability, and the effect sizes of the variants themselves are typically small. Some of the missing heritability appears to arise from common variants whose effect sizes are too small to detect using GWAS, even when the sample size is large (Boyle et al., 2017). Furthermore, the experiments required to functionally validate the role of a specific variant are resource-intensive, and the number of potentially important variants that emerge from genetic studies vastly exceeds the capacity and resources available to evaluate them.

This paper details an approach to begin to meet this need by integrating data sources across multiple experimental types to reliably detect weak genetic signals related to Parkinson’s disease (PD). Our approach probabilistically classifies genes as belonging to a null group, a deleterious group, or a beneficial group using joint models of the relationship of disparate data types to disease status. In this framework, genes in the null group are unassociated with the disease, genes in the beneficial group are associated with either a better outcome or decreased incidence of a negative outcome, and genes in the deleterious group are associated with a worse outcome or increased incidence of a negative outcome. Prior probabilities of group assignments are apportioned with a Dirichlet distribution, and thus posterior group probabilities automatically account for the multiplicity inherent in analyzing many genes simultaneously (Scott and Berger, 2010).

We consider a pair of experiments in this paper: an RNA-seq study in which biological samples of PD and control patients are assayed for differential expression, and a GWAS study where PD and control populations with no familial relationships are genotyped. Each data type has its own separately specified sub-model in which the response is modeled as conditionally independent given the group labels for each gene. These group labels are shared across both experiments. Conditional independence allows additional data types to easily be added when they become available and does not require the data types to be observed on a common set of individuals. Any number of data modalities can be combined in a single coherent probability model, as long as each collection of experimental outcomes can be formalized conditionally upon latent group labels, allowing information sharing across experiment types. Let $\mathbf {Y}_1,\dots , \mathbf {Y}_M$ be response vectors for *M* experimental data types, $\boldsymbol {\theta }= (\boldsymbol {\theta }_1^{\mathrm{T}}, \ldots , \boldsymbol {\theta }_M^{\mathrm{T}})^{\mathrm{T}}$ the model parameters corresponding to sub-models for the *M* experimental types, and $\mathbf {G}$ the shared group labels. Conditional independence allows the full data likelihood to be factorized as $\mathcal {L}(\mathbf {Y}_1,\dots ,\mathbf {Y}_M \, |\, \mathbf {G}, \boldsymbol {\theta }) = \prod _{m=1}^M\mathcal {L}_m(\mathbf {Y}_m \, |\, \mathbf {G},\boldsymbol {\theta }_m).$

The statistical framework we propose enhances the power to detect weak signals by borrowing strength across different sources of information. This approach differs from the combination of multiple datasets or studies with meta-analyses. Traditional meta-analysis approaches for integrating data sources perform separate analyses for each data type and then combine the resulting summary measures like *P*-values or odds ratios (see, e.g. Zeggini and Ioannidis, 2009 for a review of meta-analyses in GWAS studies). Many techniques for *P*-value combination exist, including Fisher’s or Stouffer’s techniques (Fisher, 1929; Stouffer et al., 1949) as well as more modern approaches (Genovese and Wasserman, 2004; Benjamini and Heller, 2008). A critical weakness of meta-analysis is that the analysis of each individual dataset is siloed and, therefore, information can only be shared through the summary measures (e.g. P-value) rather than being shared among full datasets. Finally, traditional meta-analysis can suffer from selection bias, inconsistent analytic approaches, etc. (Begum et al., 2012). In contrast, our proposed approach allows for consistent analytic treatment of each study and shares information across all data types to inform the analysis, increasing power to detect weak signals.

Several previous studies have emphasized the benefits of analyzing different datasets jointly, a strategy sometimes referred to as meta-dimensional methods (Ritchie et al., 2015) or multi-modal analysis (see Richardson et al., 2016; Li et al., 2018, for reviews). However, previous approaches to integrating multiple genomic data types are not directly applicable to our PD analysis, as they require either the same subjects in each experiment type, the same experiment types, or *a priori* grouping of genes into sets (see, e.g. Tyekucheva et al., 2011; Holzinger et al., 2013; Ding et al., 2022, and references therein).

Our hierarchical three-groups structure results in parsimonious inference with few false positives, while simultaneously enhancing power to detect signals. An additional benefit is that, like many hierarchical Bayesian formulations, it accommodates the situation where some genes are not measured in all data types. Genes that are included in some, but not all, experiment types are treated as missing data by iterative sampling from their posterior predictive distributions in the experiments from which they are absent. This flexibility is not available, for example, to *P*-value combination approaches which require genes to be measured in all data types.

Our primary scientific interest lies in the posterior probabilities of the group assignments. Genes with high posterior probability of being either beneficial or deleterious will be considered targets for our follow-up experiments, potentially as therapeutic targets.

## 2 Model details

The proposed three-groups suite of statistical models requires the separate development of a sub-model for each experimental data type, conditional on the collection of genes belonging to null, deleterious, or beneficial groups. Each sub-model is tailored to its particular response type, but each is built upon the common three-groups structure, allowing pooling of information through the shared group labels. The shared group labels $G_j$ for gene $j \in 1, \ldots J$ are modeled with a Dirichlet-categorical distribution, which induces an automatic multiplicity adjustment (Scott and Berger, 2010) (Section 2 of the Supplementary Materials). Our complete model, including the RNA-seq sub-model, the GWAS sub-model, and shared components, is written out in the first section of the Supplementary Materials.

### 2.1 three-groups model for RNA-seq data

RNA-seq expression levels are measured as counts, necessitating statistical approaches that either model the data using discrete distributions such as Poisson or Negative Binomial, like edgeR (Robinson et al., 2010) and DESeq2 (Love et al., 2014), or normalize the data before applying statistical models for continuous responses like limma+voom (Law et al., 2014). In either case, the goal of the analysis is to discover genes that are differentially expressed between the treatment (disease) and control (healthy) groups.

Let $Y_{ijk}^{\mathrm{RNA-seq}}$ be the count for the $i{\mathrm{th}}$ replicate of the $j{\mathrm{th}}$ gene for treatment group $k\in \lbrace 0,1\rbrace$. Similarly to edgeR, we model $Y_{ijk}^{\mathrm{RNA-seq}}$ using the Negative Binomial distribution, using a mean and dispersion parametrization: $Y_{ijk}^{\mathrm{RNA-seq}}\sim \mathrm{NegBin}(\mu _{ijk}, \phi _{j}),$ where $\mu _{ijk}$ is the mean count from individual *i* of gene *j* from treatment group *k*, and $\phi _j$ is the dispersion parameter for gene *j*. The variance of $Y_{ijk}$ is then $\mu _{ijk}(1+\mu _{ijk}\phi _{j})$. We model the mean counts as


\begin{eqnarray*}
\log (\mu _{ijk}) = \alpha _j + \log (fc)_j*k + L_i + M_j + (\mathbf {X}_i^{\mathrm{RNA-seq}})^{\mathrm{T}}\boldsymbol {\beta }^{\mathrm{RNA-seq}}.
\end{eqnarray*}

Here $\alpha _j$ is the gene-wise intercept, $(fc)_j$ is the fold change between expected counts in the treatment group compared to the control group, $L_i$ and $M_j$ are the natural logarithm of the library size for sample *i* and the gene length for gene *j*, respectively (known), and $(\mathbf {X}_i^{\mathrm{RNA-seq}})^{\mathrm{T}}$ is a row-vector of covariates associated with individual *i* from the RNA-seq study. When $k=0$, indicating the control group, the mean function simplifies to $\log (\mu _{ijk}) = \alpha _j + L_i + M_j + (\mathbf {X}_i^{\mathrm{RNA-seq}})^{\mathrm{T}}\boldsymbol {\beta }^{\mathrm{RNA-seq}}.$ Expected counts are proportional to both library size and gene length, in both groups, and thus these offsets are directly included in the model. This is an alternative to normalizing the data in a pre-processing step, which is common in standard analysis tools (Robinson et al., 2010; Law et al., 2014; Love et al., 2014).

The fold change $(fc)_j$ of the counts associated with each gene is the focus of the inference, and hence is endowed with the three-groups structure:


(1) \begin{eqnarray*}
\log (fc)_j\sim \left\lbrace \begin{array}{@{}l@{\quad }l@{}}\delta _0 & \mathrm{if}\ G_j=1 \text{ (Null) }\\f^{\mathrm{RNA-seq}^+} & \mathrm{if}\ G_j=2\text{ (Deleterious) }\\f^{\mathrm{RNA-seq}^-} & \mathrm{if}\ G_j=3\text{ (Beneficial)}\\\end{array}\right.
\end{eqnarray*}

where $\delta _0$ is a point mass at zero, $f^{\mathrm{RNA-seq}^+}$ is a distribution over the positive half-line, and $f^{\mathrm{RNA-seq}^-}$ is a distribution over the negative half-line (see Section 2.3).

We induce shrinkage on the gene-wise dispersion using a shared random effect scheme, with $\log (\phi _j) \sim \mathrm{N}(\mu _{0}, \mathrm{precision}= \tau _0)$, $\mu _0 \sim \mathrm{N}(0, \mathrm{precision}= 10^{-2})$, and $\tau _0 \sim \mathrm{t}^+(\nu = 4)$, where $\mathrm{t}^+$ refers to a *t*-distribution truncated to the positive half-line as in Gelman (2006).

To complete the model, we assign the vague priors $\alpha _j \sim \mathrm{N}(0, \mathrm{precision}= 10^{-3})$ and $\beta ^{\mathrm{RNA-seq}}_q \sim \mathrm{N}(0, \mathrm{precision}= 10^{-3})$, independently for $j = 1, \ldots , J$ and $q = 1, \ldots , Q^{\mathrm{RNA-seq}}$, where $Q^{\mathrm{RNA-seq}}$ is the number of covariates considered.

### 2.2 three-groups model for GWAS data with binary outcomes

GWAS entails collecting genotypic (i.e. SNVs) and phenotypic data (disease status) from unrelated individuals with the goal of associating specific SNVs with disease status. In our model formulation for the RNA-seq data type, the unit of measurement is the gene; however, GWAS data are collected on the SNV level. Thus, integrating GWAS with the other models requires that SNVs be either summarized into their associated genes or identified as non-coding variants with no obvious parallel in our RNA-seq dataset. The need to aggregate SNV information to the gene level is not unique to our formulation and is also necessary, for example, for burden tests for association (Morgenthaler and Thilly, 2007; Li and Leal, 2008; Madsen and Browning, 2009; Asimit et al., 2012). How best to formalize the association between SNVs and genes is an open question (see, e.g. Gazal et al., 2022 and references therein). The simplest approach, which we take here, is to collapse SNVs into genes in a binary fashion: if a gene contains an SNV in or near its coding region, it is assigned a value of 1; if it does not contain an SNV, it is assigned a value of 0. This binary collapse assumes a dominant model of disease (i.e. having one or more copies of the associated minor allele alters the risk). A simple alternative strategy is to sum the number of minor alleles in or near each gene’s coding region. We report results from this alternative sum-mapping in Section 6 of the Supplementary Material. Extensions include using weighted sums, and schemes allowing for different SNVs in a single region to act in opposite ways on the outcome. Our model is agnostic with respect to the particular SNV-to-gene mapping, as different mappings only require corresponding changes to the design matrix $\boldsymbol{X}_i^{GWAS}$ and thus the model itself is immediately applicable without modification.

Our three-groups model for GWAS is logistic regression, as the response is binary (1 if individual *i* in the GWAS study has PD and 0 otherwise). The response $Y_i^{\mathrm{GWAS}}$ is modeled as a Bernoulli random variable with probability $p_i$ of being in the PD group, with


\begin{eqnarray*}
\mathrm{logit}(p_i) = \mathbf {z}_i^{\mathrm{T}}\boldsymbol {\gamma }+ (\mathbf {X}_i^{\mathrm{GWAS}})^{\mathrm{T}}\boldsymbol {\beta }^{\mathrm{GWAS}},
\end{eqnarray*}

where $\boldsymbol {\gamma }= (\gamma _1, \ldots , \gamma _J)^{\mathrm{T}}$ are gene effects and $\boldsymbol {\beta }^{\mathrm{GWAS}}$ is a vector of individual-level covariate effects. Here $\mathbf {z}_i = (z_{i1}, \ldots , z_{iJ})^{\mathrm{T}}$ is a binary vector that indicates whether there was a SNV in genes $j= 1, \ldots , J$ for individual *i* and $\mathbf {X}_i^{\mathrm{GWAS}}$ is the vector of covariates from individual *i*. The gene effects are the focus of the inference, and thus the $\gamma _j$s, $j=1, \ldots , J$, are endowed with a three-groups prior structure analogous to (1) from Section 2.1:


\begin{eqnarray*}
\gamma _j\sim \left\lbrace \begin{array}{@{}l@{\quad }l@{}}\delta _0 & \mathrm{if}\ G_j=1 \text{ (Null) }\\f^{\mathrm{GWAS}^+} & \mathrm{if}\ G_j=2\text{ (Deleterious) }\\f^{\mathrm{GWAS}^-} & \mathrm{if}\ G_j=3\text{ (Beneficial)}.\\\end{array}\right.
\end{eqnarray*}

where $\delta _0$, $f^{\mathrm{GWAS}^+}$, and $f^{\mathrm{GWAS}^-}$ are defined similarly to $\delta _0$, $f^{\mathrm{RNA-seq}^+}$, and $f^{\mathrm{RNA-seq}^-}$ in section 2.1. The vague prior $\beta ^{\mathrm{GWAS}}_q \sim \mathrm{N}(0, \mathrm{precision}= 10^{-3})$ for the individual level covariates $q = 1, \ldots , Q^{\mathrm{GWAS}}$ completes the model.

### 2.3 Gene effect priors

Secondary scientific interest, after group assignment probabilities, lies in the magnitude of gene effects. These gene effects inform the group assignments and contain information regarding the potential benefit of clinical interventions. The three-groups framework allows for flexible modeling of these effect sizes through selection priors, which may be asymmetric. That is, a point mass ($\delta _0$) at zero represents the null effect, and the distribution of beneficial effect sizes ($f^{(m)^-}$) may differ from that of deleterious effect sizes ($f^{(m)^+}$). This added flexibility above standard, symmetric, selection priors reflects the biological reality that genes with protective effects may behave very differently than genes with damaging effects.

Discontinuous priors which include null and non-null components (frequently termed “spike and slab” priors) can be broadly categorized as *local* and *non-local* (Johnson and Rossell, 2010). A prior is said to be local if the density for its non-null component is non-zero in a neighborhood of the null value (i.e. very small effect sizes have prior mass). Conversely, a non-local prior has density values in its non-null component of exactly zero in a neighborhood of the null value. Intuitively, the benefit of non-local priors is that effects that might otherwise be estimated to be trivially small get pushed into the null group because very small effects have zero prior probability. We consider a non-local prior and compare the results to the local three-groups model, which has half-normal priors for the non-null gene effects.

The non-local prior that we consider is a modification of the product inverse moment (piMOM) prior from Johnson and Rossell (2010), defined by the density $f(\boldsymbol {\beta }| \tau , r) = [\tau ^{r/2} / \Gamma (r/2)]^J$  $\prod _{j=1}^J|\beta _j|^{-(r+1)} \exp {(-\tau / \beta _j^2)}$. This is a two-parameter family, with *r* controlling the tail decay (smaller *r* gives heavier tails) and $\tau$ controlling the scale. To allow for asymmetry in the non-null gene effects, we truncate the density and use the positive component that has support on $\mathbb {R}^+$ for deleterious gene effects, and separately use a negative component that has support on $\mathbb {R}^-$ for the beneficial gene effects. Separate half-piMOM hyper-priors are placed on each of the $\tau$ parameters of these truncated densities. This allows us to consider the separate posterior distributions of beneficial and deleterious gene effects. Together with a point mass at zero, we thus arrive at a three-component mixture for the null, beneficial, and deleterious gene effects. Previous work on variable selection tasks using non-local priors (Johnson and Rossell, 2010; 2012; Nikooienejad et al., 2016; Li and Chekouo, 2022, e.g) found improved performance when using non-local priors relative to standard local selection priors. Johnson and Rossell (2012) demonstrated that Bayesian model selection procedures based on the piMOM prior density with $r\ge 2$ results in consistent estimation of the true model. Here, we follow their recommendation and fix the tail decay rate at $r = 2$.

## 3 Computation

The analysis was performed using reversible jump Markov chain Monte Carlo (RJMCMC; Green, 1995), which improves runtime by accounting for the change of dimensionality when group assignment changes. A simpler conventional sampler without RJMCMC is possible, but suffers from poor mixing because it requires needlessly sampling from gene effects that will just be multiplied by zero (as most genes are null). RJMCMC avoids much of this wasted effort and is appreciably faster in our NIMBLE implementation (de Valpine et al., 2017; 2023). Details on the RJMCMC are in Section 3 of the Supplementary Materials, and code is available in the Supplementary Materials.

## 4 Simulations

In this section, we explore the behavior of our three-groups framework with asymmetric non-local gene effects relative to a local three-groups model with symmetric gene effects and standard analysis pipelines. Although we tried to replicate key features of real genomic data in our simulated datasets, the simulated data are illustrative only; thus, the total number of simulated genes is small ($J=250$) to allow for uncluttered visualizations. In each scenario, we assigned five genes to have beneficial effects and five genes to have deleterious effects. Each of the 300 simulated datasets includes a GWAS dataset and an RNA-seq dataset, which are independent conditional on the shared gene labels. To reflect the sample sizes in our actual datasets, we simulated 1000 individuals for GWAS and 100 individuals for RNA-seq. Section 4 of the Supplementary Materials contains a full description of the simulated data.

**Figure 1:**
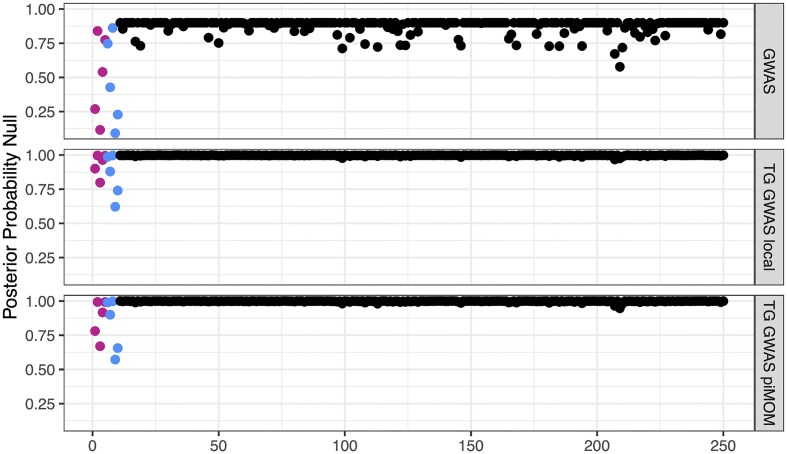
Posterior probability of inclusion in the null group for GWAS-only methods, from a single simulated dataset. The top plot shows the individual logistic regression results, the middle plot shows our three-groups GWAS model with local priors on gene effects, and the bottom plot shows our three-groups GWAS model with piMOM priors on gene effects. Genes in blue were simulated to be deleterious, genes in purple were beneficial, and genes in black were null. Abbreviations: TG, three-groups; GWAS, genome-wide association studies; piMOM, product inverse moment.

**Figure 2:**
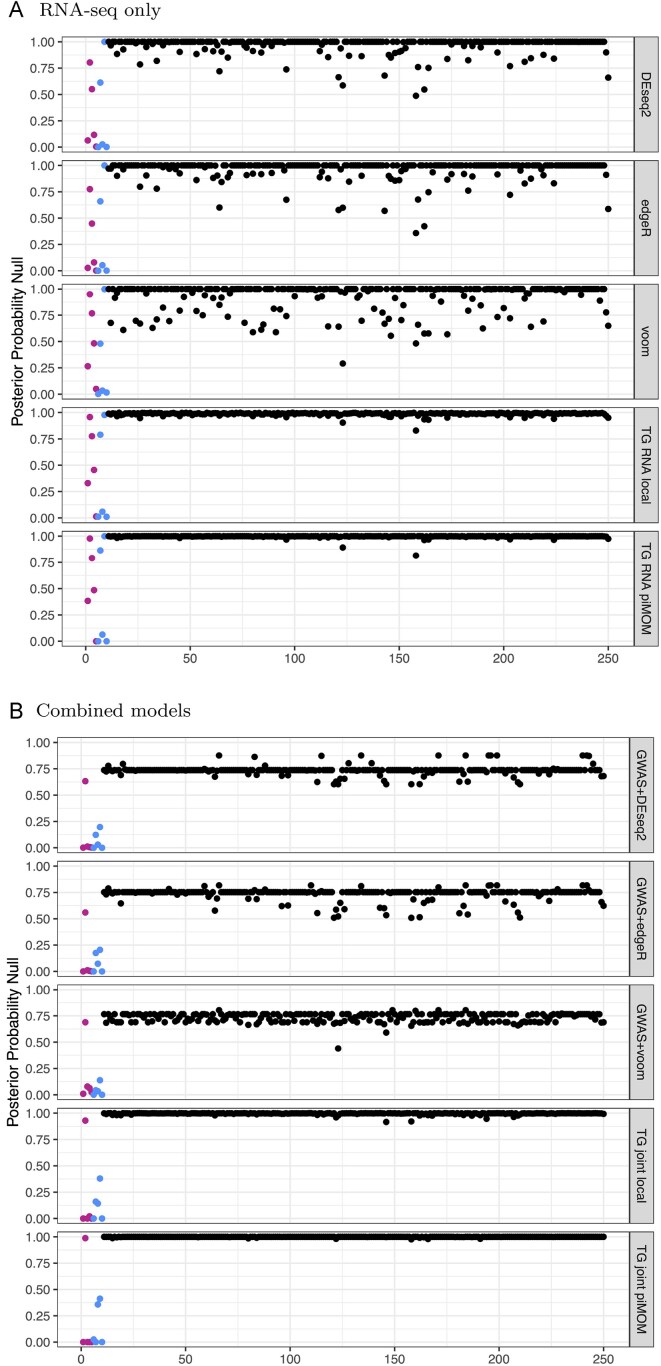
Panel (A) shows posterior probabilities of inclusion in the null group for RNA-seq only methods, from a single simulated dataset. Panel (B) shows the same, but for joint GWAS and RNA-seq methods. The top three plots in each panel are results from standard analysis tools, and the bottom two plots in each panel are results from our local and non-local three-groups models. The color scheme is the same as in Figure 1. Abbreviation: GWAS, genome-wide association studies.

We use each of the 300 simulated sets of joint GWAS and RNA-seq data to compare conventional methods, a local symmetric three-groups model, and a non-local asymmetric model for GWAS alone (Figure 1), RNA-seq alone (Figure 2a), and joint models (Figure 2b). In the joint three-groups models, the GWAS and RNA-seq models are combined into a single hierarchical model as described in the supplement. The conventional methods are combined using Fisher’s P-value combination method (Fisher, 1929) (the Cauchy combination method of Liu and Xie (2020) is explored in Section 5.2 of the Supplementary Materials). We chose competitor models for comparison based on what we take to be the most common analysis pipelines in the literature. Specifically, we used individual logistic regression (i.e. one logistic regression per gene, independently across genes) for GWAS; DESeq2, edgeR, and limma+voom for RNA-seq. Competitors’ methods were run with their default settings (including normalization) from the standard packages on Bioconductor.

We control for multiple comparisons in the conventional methods using the local false discovery rate (lFDR) (Efron et al., 2001). We chose lFDR over similar adjustment methods because of the comparable interpretation to posterior probabilities that result from Bayesian selection procedures. Specifically, the lFDR can be interpreted as the probability of a gene belonging to the null group conditional on the value of its test statistic. Additional comments concerning lFDR (including the odd clustering around 0.75 in Figure 2b) are given in Section 5.3 of the Supplementary Materials.

As an illustration, Figures 1 and 2 show the posterior probability of inclusion in the null group for each gene, from a single simulated dataset. Each dot represents a gene, and its height represents the posterior probability of being in the null group. The colored dots are true non-null genes, and the black dots are true null genes. The three-groups model further generates posterior probabilities of being in the beneficial and deleterious groups (not shown). These figures give some idea of how well each of the methods separates null from non-null genes. We investigate this more formally by considering performance across the 300 simulated joint datasets. Boxplots of run times for individual simulations are given in Section 5.8 of the Supplementary Materials.

**Figure 3:**
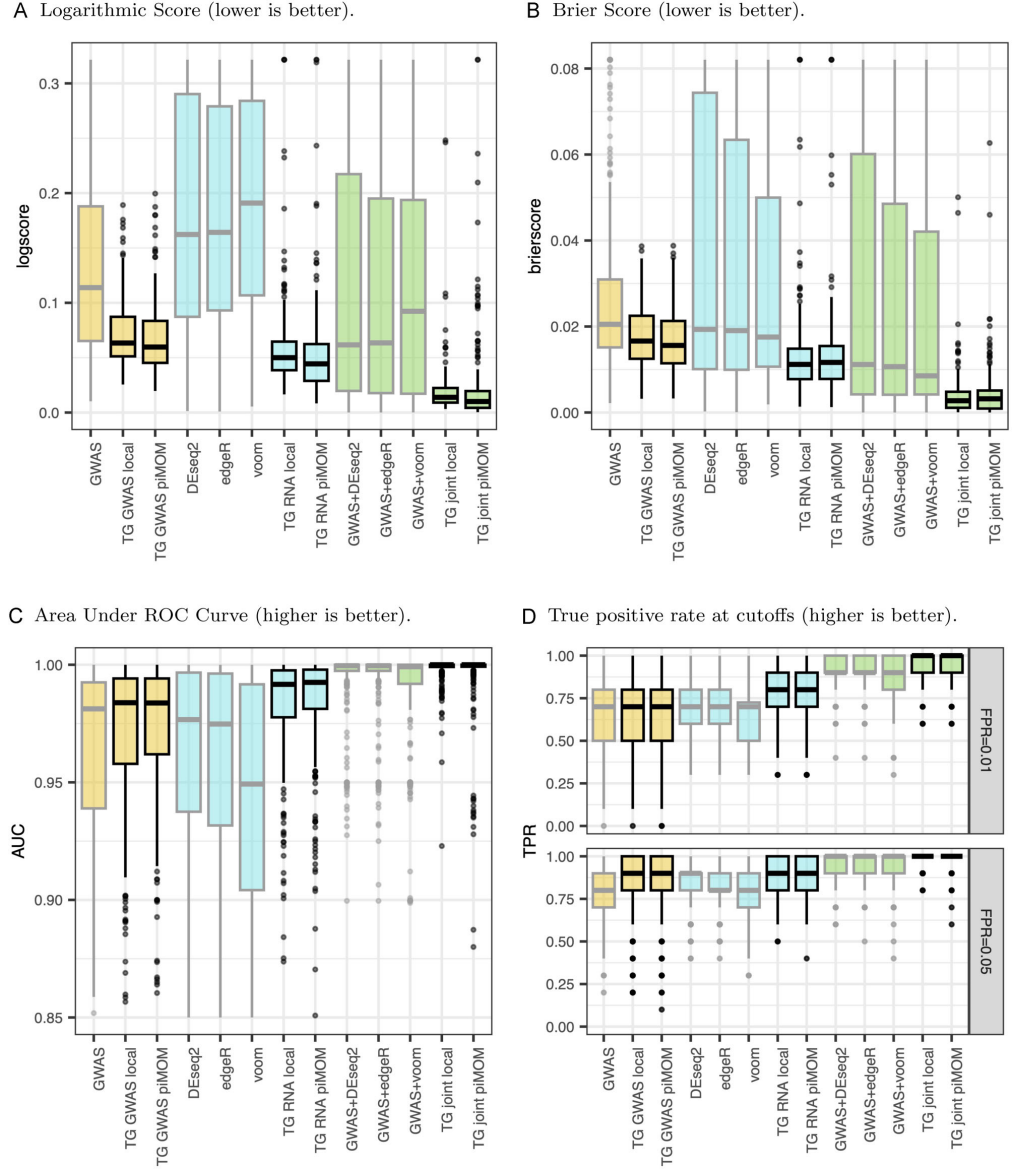
Boxplots of logarithmic scores (A), Brier scores (B), area under receiver operating characteristic curve (C) computed from posterior probability of inclusion in the null group, and the true positive rates (i.e. power) computed at classification cutoffs that result in mean false positive rates of 0.01 (top panel) and 0.05 (bottom panel) in (D) for each of 13 different models, based on 300 simulated datasets. Yellow (the first three boxes) indicates GWAS-only models, blue (the next five boxes) indicates RNA-seq only models, and green (the last five boxes) indicates joint models. Boxplots for our three-groups models have black lines, while competitors have grey lines. Panels (A), (B), and (C) show Winsorized boxplots to focus on the bulk of the distribution of points. Abbreviation: GWAS, genome-wide association studies.

We assess the simulations using a variety of performance metrics. Each metric is computed on each model for each simulated dataset, and then the models are compared using boxplots of those metrics (further discussion of the metrics is in Section 5.1 of the Supplementary Materials). This allows us to consider the performance of each model across the many simulated datasets, which aids in understanding variability. First, we consider two proper scoring rules with attractive properties: the log score and Brier score (Gneiting and Raftery, 2007), oriented so that smaller values correspond to better performance (Figure 3). Both reward correct classifications more when they are made with higher confidence, and conversely penalize incorrect classifications more when they are made with higher confidence.

The results in Figure 3 suggest that the three-groups versions of GWAS and RNA-seq, taken separately, are at least as good as standard analysis tools, and, in some cases, much better. Particularly striking is how much better the three-groups RNA-seq model performs in terms of log score, relative to DESeq2, edgeR, and limma+voom. This, again, is obtained without any normalizing schemes or elaborate tuning in the three-groups model.

To assess the performance of models at differing cutoffs, we compare the area under the receiver operating characteristic curve (Figure 3(c)), a popular metric in the classification literature. In addition, we show the true positive rate (i.e. power) computed at a cutoff for each model set such that each resulted in a fixed average false positive rate (Figure 3(d)). The joint models are superior to individual models in each metric, and our three-groups family of models correctly identifies at least as many non-null genes as competitors while including very few false positives.

These simulations (and others) demonstrate the value in the joint structure of our model (even when most of the signal comes from one data type). We investigate the performance of our model under scenarios with missing data, signal in one but not both data modalities, hyperprior sensitivity, and so forth, with additional simulations in Section 5 of the Supplementary Materials. Finally, a recurring theme in our investigation was the sparsity imposed by the three-groups family of models; Figures 1 and 2 demonstrate that the three-groups models have a strong preference for fewer non-null genes, as explained in Section 2 of the Supplementary Materials, even with a uniform (i.e. non-informative) prior on the unit simplex for the group inclusion probabilities.

## 5 PD analysis

### 5.1 Description of the data

The data that we used for the GWAS branch of this study came from the International Parkinson’s Disease Genomics Consortium NeuroX Dataset (Nalls et al., 2014). The RNA-seq data that we used came from The Parkinson’s Progression Markers Initiative (PPMI, 2018), obtained from PPMI upon request. Due to the intense computational burden of running the full MCMC, we analyzed a subset of the full genome (1734 genes in the GWAS branch and 1697 in the RNA-seq branch). Details on the data and subsetting can be found in Section 7.1 of the Supplementary Materials.

### 5.2 Description of the analysis

We analyzed the aforementioned data with the full joint three-groups model as well as with the individual sub-models separately, with both symmetric local and asymmetric non-local gene effect priors. These six models allow for comparisons between our joint and individual models, as well as comparisons between the local and non-local versions of the models. In each case, we ran the MCMC for 20 000 iterations and threw out the first 10 000 as burn-in, as indicated by trace plots.

### 5.3 Results

One of the benefits of the three-groups family of models is the sparsity it induces due to the built-in multiplicity adjustment, in addition to the borrowing of strength across both genes and sub-models. The induced sparsity is evident in this analysis: more than 1650 of the 1734 genes in the full analysis have a posterior probability of being null, which is greater than 0.99. We expect that, operationally, the *x* genes with the smallest posterior probability of being null will be investigated further in mouse models or human iPSC cell models by knocking down the genes using siRNA or CRIPSRi/a, etc., where *x* is determined by available resources such as budget and personnel time. Here, we report “interesting” genes by setting $x = 20$. An alternative would be to classify genes according to their posterior probabilities, but this necessitates the somewhat arbitrary selection of a cutoff value. Instead, we report the 20 most interesting genes alongside the posterior probabilities and gene effect sizes (Table 1).

**Table 1:** Posterior probabilities and effect sizes for the 20 genes with the smallest posterior probability of inclusion in the null group in the joint three-groups models.

	Gene	local	piMOM	GWAS + edger	GWAS + limma	GWAS + DESeq2	$P_{\mathrm{null}}$ local	$P_{\mathrm{del.}}$ local	$P_{\mathrm{ben.}}$ local	$P_{\mathrm{null}}$ piMOM	$P_{\mathrm{del.}}$ piMOM	$P_{\mathrm{ben.}}$ piMOM	GWAS effect local	RNA effect local	Disp. local	GWAS effect piMOM	RNA effect piMOM	Disp. piMOM
1	CHCHD6	+	+	+	+	+	0.00	0.00	1.00	0.00	0.00	1.00	0.81	0.90	0.16	0.80	0.86	0.16
2	DUSP1	+	+	+	+	+	0.00	1.00	0.00	0.00	1.00	0.00	1.12	1.19	0.09	1.24	1.19	0.09
3	SYTL3	+		+	+	+	0.16	0.76	0.08	1.00	0.00	0.00	1.14	1.11	0.08	*	*	0.08
4	CDIP1	+	$\circ$	+	+	+	0.26	0.09	0.65	0.49	0.13	0.38	0.36	0.93	0.14	0.41	0.87	0.14
5	FGD4	–		+	+	+	0.61	0.27	0.12	1.00	0.00	0.00	1.02	1.13	0.06	*	*	0.06
6	TLR6	–		+	+	+	0.90	0.05	0.05	1.00	0.00	0.00	1.16	1.10	0.05	*	*	0.05
7	CNTNAP2	+	+	+			0.00	0.00	1.00	0.00	0.00	1.00	0.67	0.48	1.17	0.71	0.51	1.16
8	IFRD1	+			+	+	0.27	0.62	0.11	1.00	0.00	0.00	1.03	1.13	0.07	*	*	0.07
9	RAP1GAP	–		+		+	0.85	0.06	0.09	1.00	0.00	0.00	0.96	0.59	1.54	*	*	1.53
10	FAM49B	+	+				0.00	1.00	0.00	0.00	1.00	0.00	2.19	1.06	0.02	8.67	1.09	0.02
11	DPP10	–	+				0.58	0.29	0.13	0.00	1.00	0.00	2.20	1.15	3.08	2.56	1.23	3.06
12	FCGR2A		+		+		1.00	0.00	0.00	0.00	1.00	0.00	*	*	0.04	1.28	1.13	0.07
13	CD180	–					0.85	0.06	0.08	1.00	0.00	0.00	0.88	0.84	0.17	*	*	0.17
14	PLA2G16	–					0.87	0.05	0.07	0.83	0.07	0.10	0.56	0.68	25.24	0.64	0.73	1.06
15	LINC00969	–					0.89	0.05	0.06	0.88	0.06	0.06	1.62	1.67	25.31	1.38	1.14	1.08
16	TMEM55A	–					0.89	0.05	0.06	0.87	0.06	0.06	1.65	1.73	25.06	106.49	1.07	1.08
17	MRPS21	–					0.89	0.05	0.06	0.87	0.06	0.06	1.67	1.85	25.03	1.50	1.12	1.01
18	C19orf70	–					0.89	0.05	0.06	0.87	0.07	0.06	1.54	1.63	25.36	1.45	1.13	0.99
19	C19orf66	–					0.90	0.05	0.05	0.87	0.07	0.06	2.41	2.74	25.17	1.91	1.42	1.12
20	C16orf52	–					0.90	0.05	0.05	0.88	0.06	0.06	1.61	1.65	24.94	1.46	1.18	1.01
21	ATP5J	–					0.90	0.05	0.05	0.88	0.06	0.06	1.03	1.28	25.05	1.07	1.04	1.04
22	CD82		+				1.00	0.00	0.00	0.00	0.00	1.00	1.00	1.05	0.05	0.75	0.91	0.04
23	CNTNAP4		+				1.00	0.00	0.00	0.00	0.00	1.00	0.96	0.63	3.63	0.67	0.77	4.21
24	CXCR4		+				1.00	0.00	0.00	0.00	1.00	0.00	0.94	0.86	1.30	1.55	1.10	0.05
25	DLGAP1		+				1.00	0.00	0.00	0.00	1.00	0.00	0.99	0.80	2.89	1.49	1.19	1.53
26	EFCAB6		+				1.00	0.00	0.00	0.00	0.00	1.00	*	*	0.07	0.76	0.79	1.29
27	FIGN		+				1.00	0.00	0.00	0.00	1.00	0.00	*	*	4.26	1.51	1.18	1.83
28	FRAS1		+				1.00	0.00	0.00	0.00	1.00	0.00	*	*	1.53	1.59	1.21	2.89
29	JARID2		+				1.00	0.00	0.00	0.00	1.00	0.00	*	*	0.07	1.42	1.10	0.06
30	LRFN5		+				1.00	0.00	0.00	0.00	0.00	1.00	*	*	1.84	0.67	0.77	4.46
31	PTPRN2		+				1.00	0.00	0.00	0.00	1.00	0.00	*	*	0.06	1.67	1.16	0.19
32	RIN3		+				1.00	0.00	0.00	0.00	0.00	1.00	*	*	4.52	0.71	0.86	0.26
33	VRK2		+				1.00	0.00	0.00	0.00	0.00	1.00	*	*	0.20	0.75	0.90	0.09
34	ATP8B4		+				1.00	0.00	0.00	0.44	0.13	0.44	*	*	0.26	0.74	0.91	0.07
35	C10orf90		+				1.00	0.00	0.00	0.44	0.44	0.12	*	*	0.09	1.28	1.22	3.59

The “+” symbols indicate genes that are in the top 20 most interesting genes in the respective model. The “–” symbols indicate genes that are in the top 20, even though their posterior probability of inclusion in the null group is greater than 0.5. The single gene with a “$\circ$” is not in the top 20 list for the piMOM model, but it has a posterior probability of inclusion in the null group that is less than 0.5. $P_{\mathrm{null}}$, $P_{\mathrm{del.}}$, and $P_{\mathrm{ben.}}$are the proportion of the 10 000 MCMC iterations that the gene was in the null, deleterious, and beneficial group, respectively. Effect sizes are computed conditionally (i.e. the mean of the effect size when the gene was non-null). GWAS effect sizes are odds ratios; a value less than 1 represents a protective effect and a value greater than 1 represents a damaging effect. RNAseq effect sizes are fold changes and are interpreted similarly. Gene effects with “*” indicate that the gene was null in the MCMC iterations in that model.

Our model identifies beneficial genes as well as deleterious genes (e.g. *CHCHD6* and *DUSP1* have posterior probability 1 of inclusion in the beneficial and deleterious groups, respectively, in both the local and non-local models). Five of the 20 most interesting genes from the local model are included in the 20 most interesting genes in the non-local model, and ten of the 35 genes identified by our three-groups models are in at least one of the lists of interesting genes from the *P*-value combinations of conventional methods.

Some identified genes have conflicting evidence between the two models. Some genes (e.g. *IFRD1* and *SYTL3*) have very small estimated gene effects in the local model (Table 1). These genes are not identified by the non-local model because very small effects are shrunk to the null value by design. There are other genes that do not appear to fit this pattern, and we conjecture that the differences may be attributable to interactions among genes estimated as non-null, which alter the likelihood values. Finally, some genes are harder to categorize. For example, *CDIP1* would be classified in the beneficial group in the local model (posterior inclusion probability of 0.65) while the non-local model has posterior probabilities of 0.49, 0.13, and 0.38 for the null, deleterious, and beneficial groups, respectively. The differences between models are not surprising when considering that the standard methods also report gene lists that are not consistent.

Our three-groups model identified several genes that have known links to PD in the literature. Several of the other identified genes are linked to pathways that have been implicated in PD, though we are unaware of previous work that directly links these genes to PD. Details of connections of the genes we identify to the PD literature are explored in Section 7 of the Supplement. Additional results, including trace plots (Section 8), volcano plots (Section 9), and lists of genes indicated as interesting by the separate three-groups models and conventional models (Sections 10 and 11), are in the Supplementary Material.

## 6 Discussion

Structuring models for data conditional on the three-groups framework has the advantages of modular incorporation of multiple experimental data types and automatic multiplicity adjustment. This increases power for improved prioritization of genes that show evidence of involvement in disease by combining information across genomic, transcriptomic, and potentially other data types. Furthermore, with the three-groups model as a platform, in the future, we can combine functional data from future cell-based studies and screens that directly assess the impact of a gene on a cell’s biological outcome, such as health, morphology, stress response, and proteostasis. In this way, we can harness the power of cell biology together with human genetics and gene expression. Additionally, the Bayesian formulation and use of MCMC allow for the inclusion of genes that have observations in some but not all data types.

One challenge that we faced was the computational burden of running MCMC on this model; each run on the PD dataset used around 30Gb of RAM and took several days to finish. This is not a huge problem in the context of a years-long collaboration like ours, but it is inconvenient. NIMBLE’s RJMCMC features reduced the computational time, but not enough to make the sampler practical in casual settings. Previous implementations of non-local selection priors saved time by approximately marginalizing out the effect sizes with a Laplace approximation (Johnson and Rossell, 2012; Nikooienejad et al., 2016; Shin et al., 2018). This was unappealing to us because the effect sizes are important in our context.

An alternative meta-analysis type approach uses conventional methods to estimate summary statistics and then treats those summaries as data in a hierarchical model. We adapted our model to use summary statistics by replacing the experiment-specific models with a summary model. In particular, we treat the estimated gene effects, $\widehat{\log (fc)}_j$ and $\hat{\gamma }_j$, as approximately normal centered on the true effect sizes and use our hyperprior on the true effect sizes. Preliminary assessment of this summary statistics model (Section 5.10 of the Supplement) suggests that a complete investigation (in future work) is warranted. The summary model is competitive in our standard simulation scenario but loses power, compared to our full model, with deviations from that scenario.

One issue that requires further attention is the effectiveness of our mapping between SNVs and genes in either GWAS or whole-genome sequencing (WGS) datasets, particularly for SNVs in non-coding regions. The technique that we used (Section 2.2) for mapping SNVs into genes is simplistic and could result in missed signals if SNVs within a single gene work in opposite directions. Other mapping techniques may enrich the procedure.

The strategy of combining genomic, transcriptomic, phenotypic, and potentially other sources of information using the three-groups framework can be applied to any heritable disease with multiple data types available. The PD datasets that we analyzed here are publicly available, but others, like family pedigree WGS, have been collected by our lab, and still others, including small interfering RNA screens, will result from follow-up experiments.

## Supplementary Material

ujag090_Supplemental_Files Web Appendices, Tables, and Figures referenced in Sections 2, 3, 4, 5, and 6, as well as data and code for the simulation study, are available with this paper at the Biometrics website on Oxford Academic. Code is also available at https://github.com/twixson/three_groups_simulations.

## Data Availability

The data that support the findings in this paper were provided by the International Parkinson Disease Genomics Consortium (NeuroX GWAS data) and the Parkinson’s Progression Markers Initiative (RNA-seq data, obtained upon request). Data will be shared on request to the corresponding author with permission of the International Parkinson Disease Genomics Consortium and the Parkinson’s Progression Markers Initiative.
