# Supplementary material for: A three-groups non-local model for combining heterogeneous data sources to identify genes associated with Parkinson’s disease
Source: Biometrics. 2026 May 30;82(2):ujag090. doi: 10.1093/biomtc/ujag090 (PMC13221837; doi:10.1093/biomtc/ujag090)
Supplement: ujag090_Supplemental_Files — Web Appendices, Tables, and Figures referenced in Sections 2, 3, 4, 5, and 6, as well as data and code for the simulation study, are available with this paper at the Biometrics website on Oxford Academic. Code is also available at https://github.com/twixson/three_groups_simulations. [file ujag090_supplemental_files.zip › TG_supplement_0426.pdf]

BIOMETRICS 000, 000–000 DOI: 000
000 0000

Supplementary Materials for
A Three-groups Non-local Model for Combining Heterogeneous Data Sources to Identify Genes Associated with Parkinson's Disease

Troy P. Wixson[1,*], Benjamin A. Shaby[1], Daisy L. Philtron[2],

International Parkinson Disease Genomics Consortium (IPDGC)[3],

Leandro A. Lima[4], Stacia K. Wyman[5], Julia A. Kaye[4], and Steven Finkbeiner[4,6,7]

[1]Department of Statistics, Colorado State University, Fort Collins, Colorado, U.S.A.

[2]Department of Applied Mathematics and Statistics, Colorado School of Mines, Golden, Colorado, U.S.A.

[3] https://pdgenetics.org

[4] Center for Systems and Therapeutics, Gladstone Institutes, San Francisco, CA, USA

[5]Innovative Genomics Institute, University of California Berkeley, Berkeley, California, U.S.A.

[6] Taube/Koret Center for Neurodegenerative Disease, Gladstone Institutes, San Francisco, CA, USA

[7]Department of Neurology and Physiology, University of California San Francisco, San Francisco, California, U.S.A.

**email:* twixson@rams.colostate.edu

This paper has been submitted for consideration for publication in *Biometrics*

1. Full model

Y_{ijk}^{RNA} is the RNA count from individual i for gene j.

$$Y_{ijk}^{RNA} \sim \text{NegBin}(\text{mean } = \mu_{ijk}, \text{dispersion } = \phi_j)$$
$$\log(\mu_{ijk}) = \alpha_j + \log(fc)_j * k + L_i + M_j + (\mathbf{X}_i^{RNA})^T \beta^{RNA}$$
$$\alpha_j \sim N(0, \text{precision} = 10^{-3})$$
$$\log(fc)_j \sim \begin{cases} 0 & \text{if } G_j = 1 \\ f^{RNA^+} & \text{if } G_j = 2 \\ f^{RNA^-} & \text{if } G_j = 3 \end{cases}$$
$$k = \mathbb{I}\{\text{individual } i \text{ has PD}\}.$$
$$L_i = \log(\text{library size of individual i}).$$
$$M_j = \log(\text{gene length of gene } j).$$
$$\mathbf{X}_i^{RNA} : \text{ covariates for individual i.}$$
$$\beta_q^{RNA} \sim N(0, \text{precision} = 10^{-3})$$
$$f^{RNA^+} \sim \text{half-piMOM}(t = t^{RNA^+}, r = 2)$$
$$-f^{RNA^-} \sim \text{half-piMOM}(t = t^{RNA^-}, r = 2)$$
$$t^{RNA^+} \sim \text{half-piMOM}(t = 0.05, r = 1)$$
$$t^{RNA^-} \sim \text{half-piMOM}(t = 0.05, r = 1)$$
$$\log(\phi_j) \sim N(\mu_0, \text{precision} = \tau_0)$$
$$\mu_0 \sim N(0, \text{precision} = 10^{-2})$$
$$\tau_0 \sim t^+(\nu = 4)$$

Y_i^{GWAS} is 1 if the ith individual in the GWAS study has PD and 0 otherwise.

$$Y_i^{GWAS} \sim \text{Bern}(p_i)$$
$$\text{logit}(p_i) = \mathbf{z}_i^T \gamma + (\mathbf{X}_i^{GWAS})^T \beta^{GWAS}$$
$$z_{ij} = \mathbb{I}\{\text{Individual } i \text{ has a SNV in gene } j\}$$
$$\gamma_j \sim \begin{cases} 0 & \text{if } G_j = 1 \\ f^{GWAS^+} & \text{if } G_j = 2 \\ f^{GWAS^-} & \text{if } G_j = 3 \end{cases}$$
$$\mathbf{X}_i^{GWAS} : \text{ covariates for individual i.}$$
$$\beta_q^{GWAS} \sim N(0, \text{precision} = 10^{-3})$$
$$f^{RNA^+} \sim \text{half-piMOM}(t = t^{GWAS^+}, r = 2)$$
$$-f^{RNA^-} \sim \text{half-piMOM}(t = t^{GWAS^-}, r = 2)$$
$$t^{GWAS^+} \sim \text{half-piMOM}(t = 0.05, r = 1)$$
$$t^{GWAS^-} \sim \text{half-piMOM}(t = 0.05, r = 1)$$

$$G_j \sim \text{Multinomial}\big(n = 1, p_m = (\lambda_1, \lambda_2, \lambda_3)\big)$$
$$(\lambda_1, \lambda_2, \lambda_3) \sim \text{Dirichlet}(1, 1, 1)$$

2. A Three-component Mixture and Automatic Multiplicity Adjustment

Our three-groups model adjusts for multiple comparisons by assigning a prior distribution to the vector of group assignment probabilities $\boldsymbol{\lambda}$ that depends on the number of comparisons J. The unknown group label G_j for gene j has a categorical distribution with probability vector denoted as $\boldsymbol{\lambda} = (\lambda_1, \lambda_2, \lambda_3)^{\mathrm{T}}$, exchangeably across all genes $1, \ldots, J$. The common structure across the sub-models induces sharing of information among the disparate data types. Placing a prior distribution on $\boldsymbol{\lambda}$ results in a penalty for large numbers of non-null genes that acts as an automatic

adjustment for multiple comparisons, and hence results in few false positives. This automatic multiplicity adjustment is well known in the beta-binomial case (Scott and Berger, 2010) and is an alternative to the common practice of performing many independent tests and adjusting p-values *post hoc* (Benjamini and Hochberg, 1995, e.g.).

The apportionment of prior mass on $\boldsymbol{\lambda}$ depends on the number of comparisons J and induces automatic multiplicity adjustment (Scott and Berger, 2010). Let $\mathcal{M}_{\mathbf{G}}$ be the model with group assignments $\mathbf{G} = (G_1, \ldots, G_J)$ where $G_j \in 1, 2, 3$ for all $j \in 1, \ldots, J$. Assign the hyper-prior distribution $\boldsymbol{\lambda} \sim \text{Dirichlet}(\kappa \mathbf{a})$. Define $(j_1, j_2, j_3)^{\mathrm{T}}$ as the number of genes in groups 1, 2, and 3 as determined by $\mathbf{G}$ (so that $j_1 + j_2 + j_3 = J$). Then the prior probability mass function (pmf) for each model $\mathcal{M}_{\mathbf{G}}$ given $\boldsymbol{\lambda}$ is $P(\mathcal{M}_{\mathbf{G}} \,|\, \boldsymbol{\lambda}) = \lambda_1^{J-j_2-j_3} \lambda_2^{j_2} \lambda_3^{j_3}$ and the marginal prior pmf for each model $\mathcal{M}_{\mathbf{G}}$ is

$$p(\mathcal{M}_{\mathbf{G}}) = \int_{\boldsymbol{\lambda}} P(\mathcal{M}_{\mathbf{G}} \,|\, \boldsymbol{\lambda}) \pi(\boldsymbol{\lambda}) \mathrm{d}\boldsymbol{\lambda} = \frac{\Gamma\left(\sum_{i=1}^{3} \kappa a_i\right) \prod_{i=1}^{3} \Gamma(\kappa a_i + j_i)}{\Gamma\left(\sum_{i=1}^{3} \kappa a_i + j_i\right) \prod_{i=1}^{3} \Gamma(\kappa a_i)}.$$

For simplicity of visualization, assume that $j_2 = j_3$ so that $j_2 + j_3$ is the number of non-null genes and $j_1 = J - 2 * j_2$ is the number of null genes. Also assume $\kappa = 1$, and $\mathbf{a} = (1, 1, 1)^{\mathrm{T}}$. If $J = 1,000$ genes, then the log marginal prior pmf for each model $\mathcal{M}_{\mathbf{G}}$, as a function of the number of non-null genes $j_2 + j_3$ is displayed in Figure 1.

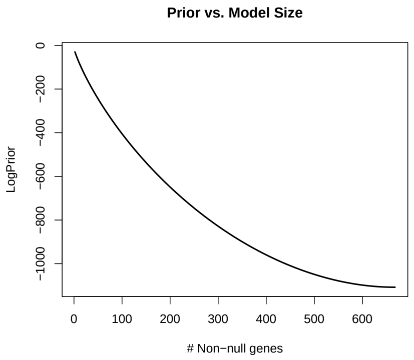

Figure 1. The log prior probability mass function for models $\mathcal{M}_{\mathbf{G}}$ corresponding to group assignments $\mathbf{G}$, as a function of the number of non-null elements in $\mathbf{G}$. The penalty for including more non-null genes in the model is strong, resulting in an aggressive multiple comparisons adjustment.

Figure 1 shows a strong prior preference for models with few genes classified as non-null. In this simple example with $J = 1000$ genes, the model with no non-null genes is many thousands of times more likely *a priori* than each model with 50 non-null genes. This strong prior preference results in posterior inference with few false positives.

To understand this prior preference, let the model size vector (j_1, j_2, j_3) denote a model with j_1 genes in group 1, j_2 genes in group 2, and j_3 genes in group 3. Observe that there are $\binom{J}{j_1, j_2, j_3}$ models of size (j_1, j_2, j_3) and thus when we add the prior mass of all models of size (j_1, j_2, j_3) we get

$$\binom{J}{j_1, j_2, j_3} \frac{\Gamma\left(\sum_{i=1}^3 \kappa a_i\right) \prod_{i=1}^3 \Gamma(\kappa a_i + j_i)}{\Gamma\left(\sum_{i=1}^3 \kappa a_i + j_i\right) \prod_{i=1}^3 \Gamma(\kappa a_i)} = \binom{J}{j_1, j_2, j_3} \frac{\Gamma(3) \prod_{i=1}^3 \Gamma(1 + j_i)}{\Gamma\left(3 + \sum_{i=1}^3 j_i\right) \prod_{i=1}^3 \Gamma(1)}$$
$$= \binom{J}{j_1, j_2, j_3} \frac{2 j_1! j_2! j_3!}{(J+2)!}$$
$$= \frac{2}{(J+1)(J+2)}.$$

Note that (1) this doesn't depend on the model size (j_1, j_2, j_3) and (2) this is the inverse of the size of the set of all possible model sizes from J genes. Finally, note that there are many more models of size $(334, 333, 333)$ than models of size $(998, 1, 1)$ and thus each individual model of size $(998, 1, 1)$ is much more likely *a priori* than each model of size $(334, 333, 333)$. Changing the Dirichlet hyper-prior values changes the specific mass but we find this explanation remains useful in understanding the sparsity inducing mechanism which relies on the prior exchangability.

2.1 *Computational Efficiency*

For computational efficiency, we use a stick-breaking representation for the Dirichlet-multinomial portion of the model. That is, for $\boldsymbol{\lambda} \sim \text{Dirichlet}(\kappa \mathbf{a})$ the marginal distribution of each component follows a Beta distribution (e.g. $\lambda_1 \sim \text{Beta}(\kappa a_1, \kappa a_2 + \kappa a_3)$) which allows us to model the prior probabilities according to two Beta distributions: one controlling the prior probability of being a null gene and the other controlling the conditional probability of being beneficial given the gene is not in the null group (Gelman, 2014, pg. 585). Correspondingly, the marginal and conditional distributions of categorical random variables are Bernoulli.

3. RJMCMC comments

Our numerical implementation used `NIMBLE`'s RJMCMC which required some additional customization. Genes in the null group have effect sizes of zero, but our numerical implementation requires beneficial and deleterious effect sizes to be updated at every MCMC iteration. In a traditional sampler, the effect sizes for genes in the null group are multiplied by an indicator of inclusion into one of the non-null groups in an intermediate step in each iteration. We used `NIMBLE`'s RJMCMC in order to cut out this unnecessary sampling and multiplication by zero. Unfortunately, the RJMCMC does not actually shrink the parameter space but instead fixes the excluded parameters at some pre-specified value (zero by default). In our case, this sets the effect sizes for null genes at zero.

Updating the hyper-parameter values (e.g., τ) requires a likelihood calculation which includes all of these fixed values. This fixing of effect sizes at zero results in errors because the non-local effect-size distributions have no mass at zero. To overcome this challenge, we wrote custom distributions which set the log-likelihood at zero for all effects which are set at zero by the RJMCMC toggler. This results in equivalent likelihood calculations to those from the true shrunk parameter space.

4. Data Generation for Simulations

We simulated GWAS datasets from a standard logistic regression model. This scenario is idealistic in that we fit the exact data-generating response model; however the standard GWAS pipeline also fits this data-generating response model, so comparisons between our three-groups approach and standard GWAS are on an even footing. To generate the binary predictor variables, which indicate the presence (or absence) of any minor alleles, we simulated a gene-wise minor allele frequency from a Beta$(20, 35)$ distribution, which has most of its mass between 0.2 and 0.5 (i.e., for $X \sim$ Beta$(20, 35)$, $P\{X \in (0.2, 0.5)\} = 0.977$). Then, conditional on the minor allele frequency, we simulated the binary predictor for each individual for that gene from a Bernoulli distribution with success probability equal to the minor allele frequency. Next, the probability of being in the treatment group is simulated from a Bernoulli distribution with success probability equal to the inverse logit of a linear combination of a normally distributed intercept and the predictors, multiplied by the fixed gene effects. The sign of the gene effects indicates group membership, with positive effects being deleterious and negative effects being beneficial.

To make things as realistic as possible, we generated the RNA-seq data from subsets of a real RNA-seq dataset. This involved selecting 250 genes from an RNA-seq dataset and adding signal to the genes which should be included in the beneficial and deleterious groups. We started with the combined data from Pickrell et al. (2010) and Montgomery et al. (2010) (`https://bowtie-bio.sourceforge.net/recount/`), as this dataset has a large number of biological replicates (129) and many genes (11,107). We added signal using a binomial thinning scheme, as in Gerard (2020), using the referenced `R` package `seqgendiff`. This method allows the simulated data to retain the characteristics of real RNA-seq data and does not bias results towards one method or another.

5. Simulation Comments and Additional Simulations

5.1 *Metrics used to compare models*

We give a few more details of the metrics that were discussed in section 4 of the main text. To compute the logarithmic and Brier scores we let p_j^{null} be the posterior probability that gene j is null and let x_j be an indicator which is 1 if gene j is null. The log score is a binomial log-likelihood and we negate it so that smaller values indicate better performance:

$$\text{log-score} = -\frac{1}{J}\sum_{j=1}^{J}\left[x_j \log(p_j^{null}) + (1 - x_j)\log(1 - p_j^{null})\right].$$

The Brier score squares the distance between the prediction and the truth:

$$\text{Brier-score} = \frac{1}{J}\sum_{j=1}^{J}(x_j - p_j^{null})^2.$$

The receiver operating characteristic curve plots the false positive rate vs the true positive rate. We report the area under this curve as this is a one-number summary of the performance of the classifier. A random classifier will have an average AUC of 0.5 and a perfect classifier will have an AUC of 1. Our final comparison metric is the true positive

(a) Logarithmic Score (lower is better). (b) Brier Score (lower is better).

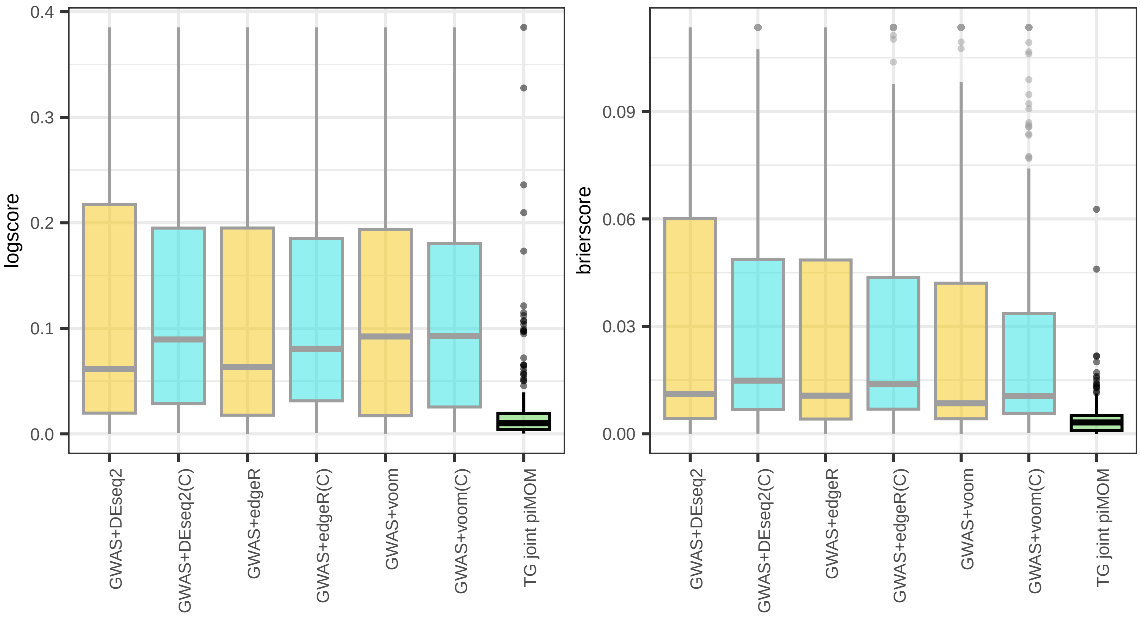

Figure 2. Comparison of p-value combination methods. Yellow filled boxplots (i.e., without "(C)") are results using Fisher's combination method and are the same as in the main manuscript. Blue filled boxplots (labeled with "(C)") are results using the Cauchy combination method was used. Included is our model for comparison (filled in green).

rate at a fixed false positive rate. For this metric we determine the cutoff at which the average false positive rate (across 300 simulations) is at the fixed value (0.01 and 0.05) and compute the true positive rate for each simulation.

5.2 *p-value Combination*

The simulation in the main text compares our joint model with p-value combinations of competitors models. There are many p-value combination methods. The plots created for the main text used Fisher's p-value combination which computes the test statistic $p_{f,comb} = -2\sum_{i=1}^{k}\log(p_i)$ where p_i is the p-value for the ith test (Fisher, 1929). This test statistic has a chi-squared distribution under the null. A different method constructs the test statistic $p_{c,comb} = \sum_{i=1}^{k} w_i \tan\{(0.5 - p_i)\pi\}$ which, under the null, can be approximated by a Cauchy distribution (Liu and Xie, 2020). Figure 2 displays the difference in simulation results for these two combination methods. These plots show that, while the particular scores are different, the character of the results is the same with both combination methods because, unlike our model, p-value combination methods cannot share information across datasets.

5.3 *lFDR Comments*

Simulation results in the main text show the lFDR when computed using the default values in the `qvalue` package. Inherent in any estimation of lFDR is an estimation of p_0 which is the true proportion of null variables (genes in our case). When we allowed the software to estimate p_0 we noticed that the posterior probability plots for competitors had most of the null genes around some value that was less than one (e.g., in Figure 2b competitors models have null genes all around 0.75). This strange artifact is do to poor estimation of p_0. We investigate the effect of this estimation

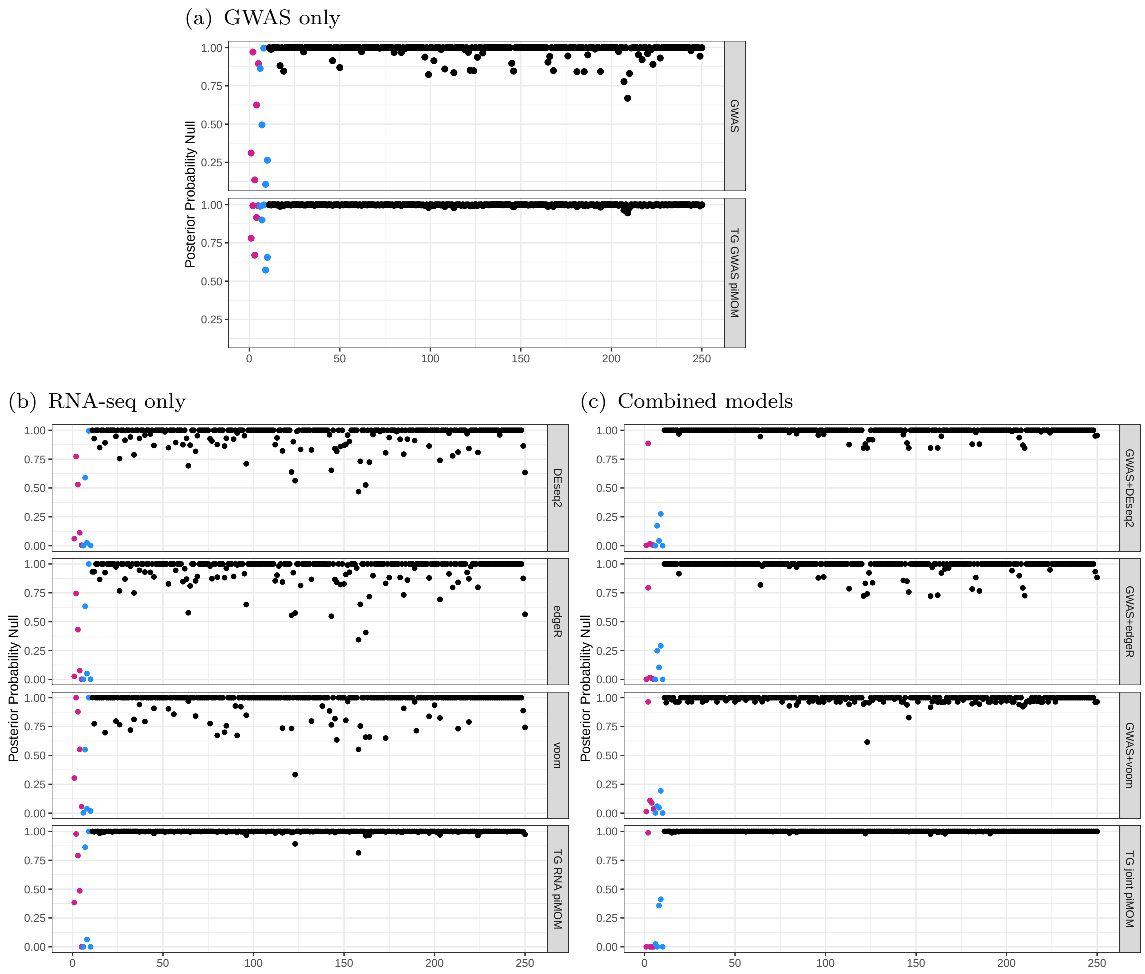

Figure 3. Posterior probability of inclusion in the null group for one simulation when true proportion of null genes is used in the LFDR computation for competitors.

by specifying the true p_0 in this section. Figure 3 shows plots analogous to Figures 1 and 2 in the manuscript. We note that now, in the competitors models, the majority of the null genes have posterior probability of inclusion in the null group of 1 which is similar to our model. While the poor estimation of p_0 alters plots of a single run quite a bit, we note that the character of the full results does not change. Figure 4 plots the boxplots of logscores and Brier scores from the same simulation as was done in the main text but with the lFDR computed with the true p_0 with both Fisher's p-value combination and the Cauchy combination method. This additional information (which is not available in a real data context) noticeably improves the scores of the competitors' methods but our model still outperforms the competitors in these metrics.

5.4 *Benefits of the Dirichlet hyperprior*

The inclusion of the Dirichlet-Multinomial hyperprior complicates our model and thus it may be useful to breifly explore the benefits. We do so by performing simulations where the probability of inclusion in a non-null group is fixed at 1/2 and 1/25 where 1/25 is the true inclusion probability. Results from these simulations are in Figure 5. These figures demonstrate that performance is similar when the inclusion probability is fixed at the truth but is substantially

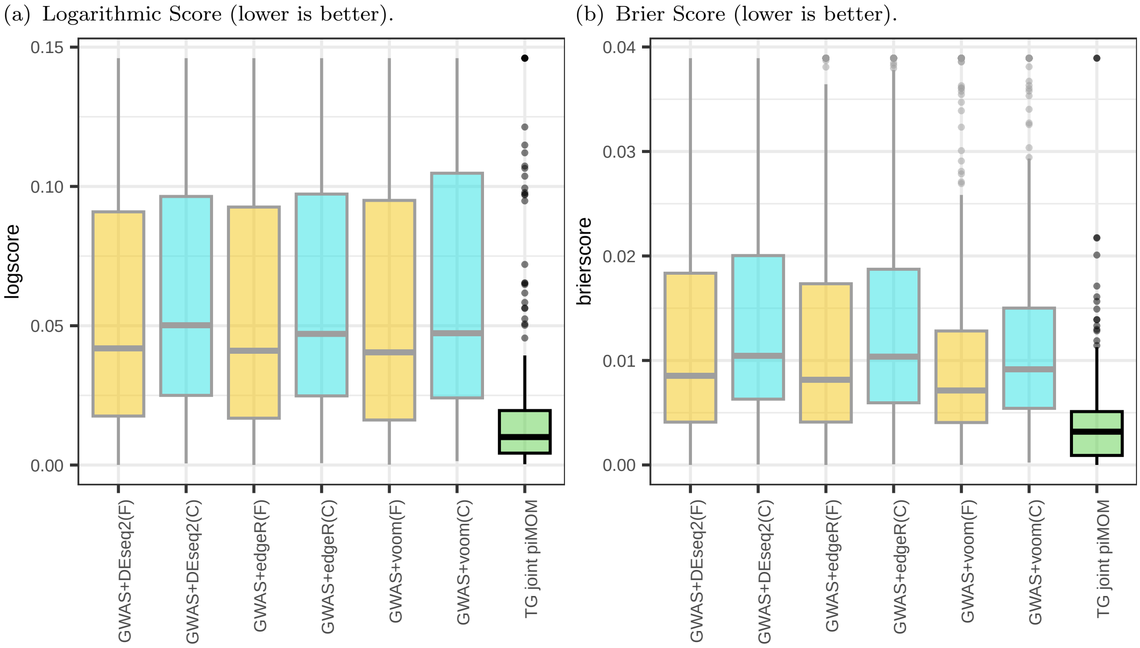

Figure 4. Comparison of models when the true proportion of non-null genes is used in the computation of the LFDF for competitors methods. Labels with "(C)" indicate that the Cauchy combination method was used (see previous section). Labels with "(F)" indicate that Fisher's combination method was used.

worse when inclusion probabilities are fixed at non-informative values. This differs from the Dirichlet-Multinomial structure where our Dirichlet hyperprior is uniform (and thus non-informative) and yet is adaptive to differing sample sizes. The adaptive nature of the Dirichlet-Multinomial structure for automatic multiplicity correction is what allows us to perform simulations with a small number of genes and suggest that performance will be similar when many more genes are included. These plots also demonstrate that, even when the inclusion probabilities are much too loose, there are benefits to borrowing strength across data types.

5.5 *Hyperprior sensitivity*

We ran several additional versions of the three-groups model and report results here. The main text includes an asymmetric non-local three-groups model which has half-piMOM effect size distributions ($f^{(m)^-}$ and $f^{(m)^+}$) with separate half-piMOM hyper-priors placed on the scaling parameter τ. This model is compared to a symmetric local three-groups model which has half-normal effect size distributions that are fixed. In this appendix we compare these models to another asymmetric non-local model that instead has inverse gamma hyper-priors on the τ parameters, a symmetric non-local model that has fixed τ values, and an asymmetric local model that has separate inverse gamma hyper-priors on the means and separate half-piMOM hyper-priors on the standard deviations of the half-normal effect size priors. Table 1 demonstrates the distinctions between these models. We do not include results from an asymmetric local model which had separate inverse gamma hyper-priors on the means and standard deviations as they were uniformly worse and made the plots unreadable.

Figure 6 demonstrates a range of choices (for modeling the effect sizes) which provide comparable results. This

(a) Logarithmic Score (lower is better). (b) Brier Score (lower is better).

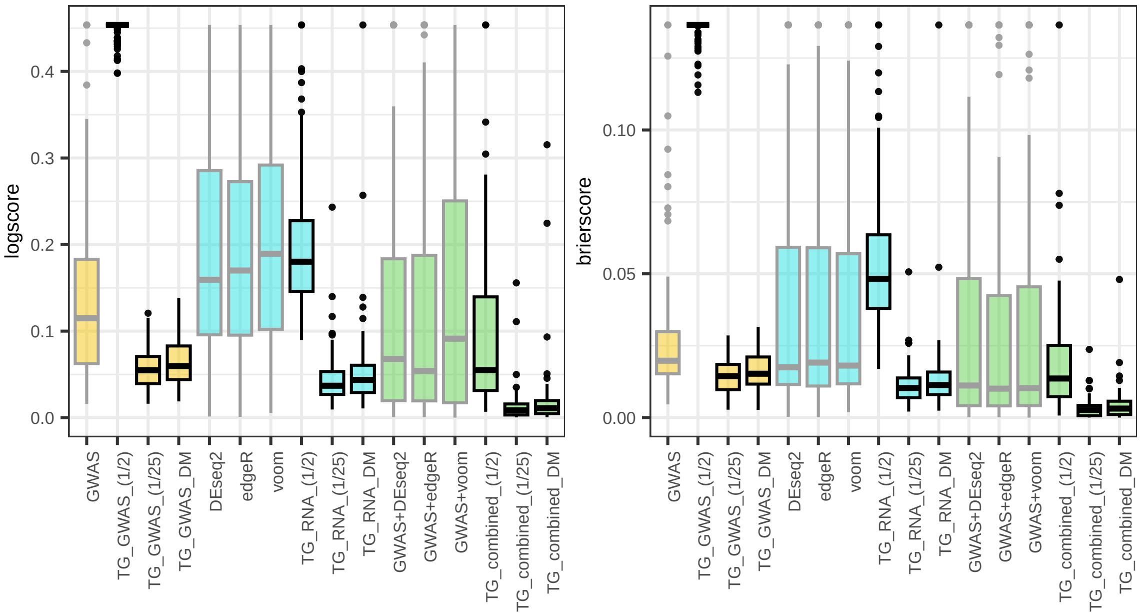

Figure 5. Comparison of models. The three TG models all have piMOM hyperpriors on gene effects but differ in the structure for inclusion in non-null groups. The first two, which are labeled with "…(1/2)" and "…(1/25)", have probabilities of being included in one of the non-null groups fixed at 1/2 and 1/25. The models labeled with "DM" have the Dirichlet-Multinomial prior setup to adaptively learn these probabilities.

Table 1
Additional three-groups models

Label	Effect size distributions	Hyper-parameters	Is local?	Is symmetric?
nonL piMOM	half-piMOM	$\tau \sim$ half-piMOM	non-local	asymmetric
nonL invG	half-piMOM	$\tau \sim$ inverse Gamma	non-local	asymmetric
nonL fixed	half-piMOM	fixed	non-local	symmetric
local piMOM	half-Normal	$\mu \sim$ inverse Gamma<br>$\sigma \sim$ half-piMOM	local	asymmetric
local fixed	half-Normal	fixed	local	symmetric

range illustrates that most of the benefit of our model comes from the borrowing of strength across data types using three-groups structure.

While there are many similarities between these plots, we think that it is worth highlighting one important difference. The non-local model with inverse gamma hyper-priors required tuning of the hyper-parameter values; we had to move the mass away from zero to ensure the posterior distributions for τ were not nearly zero. This suggests to us that the sparsity invoked by the automatic multiplicity correction of the Dirichlet-categorical inclusion scheme can become swamped by many very small empirical effect sizes. In other words, some engineering is required to ensure that the effect size distributions do not become additional "spikes" at trivially small values.

Many hyperparameters in the model are set to make the hyperpriors approximately flat in the region of interest (e.g.,

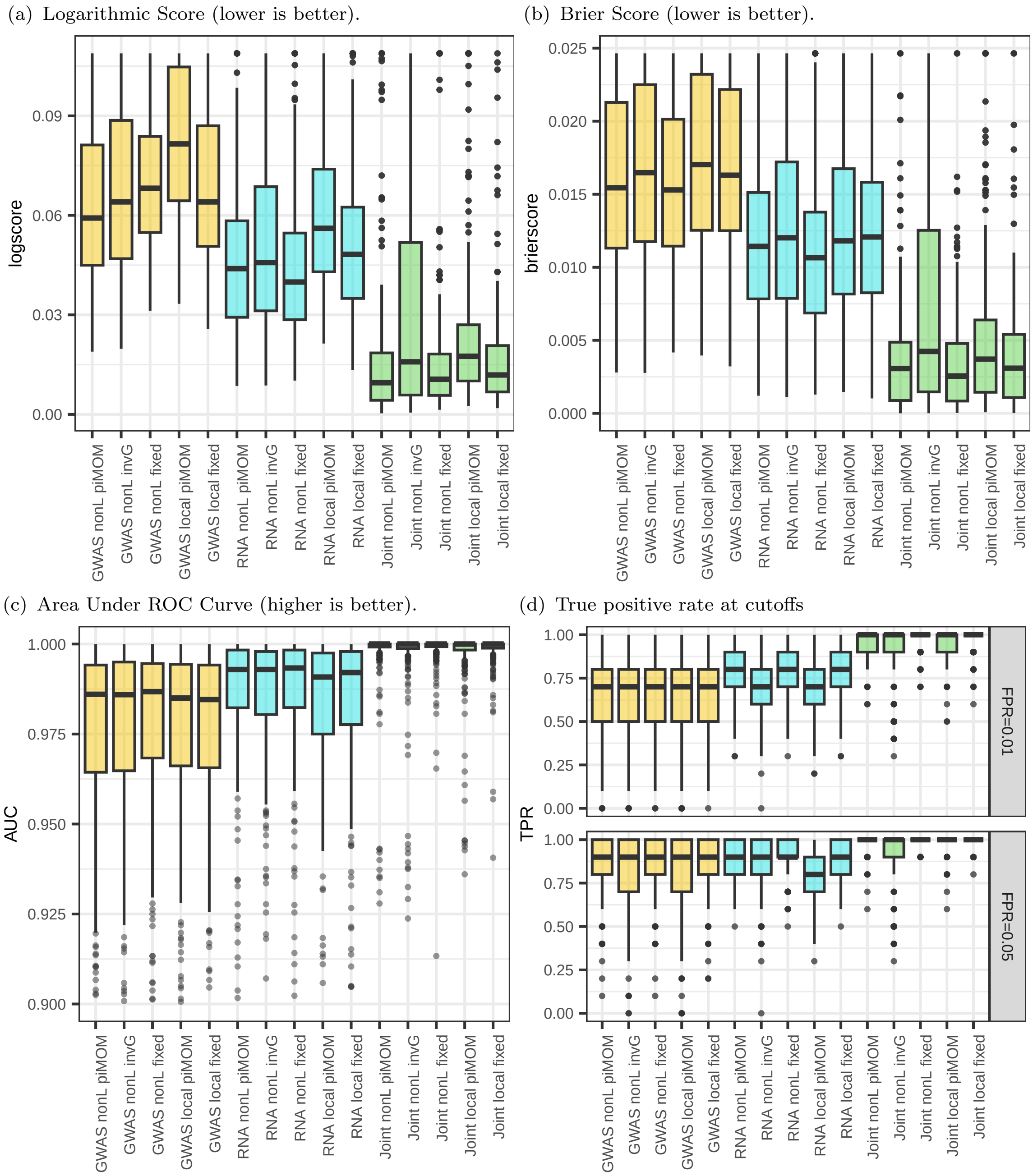

Figure 6. Boxplots of logarithmic scores (a), Brier scores (b), area under receiver operating characteristic curve (c) computed from posterior probability of inclusion in the null group, and the true positive rates (i.e. power) computed at classification cutoffs that result in mean false positive rates of 0.01 (top panel) and 0.05 (bottom panel) in (d) as in Figure 4 in the main text. The first term in the labels ("GWAS", "RNA", "Joint") indicates which sub-models are run. The second term in each label ("nonL" or "local") indicates whether the gene effect size distributions are non-local or local. the last term ("piMOM", "invG", "fixed") indicates the hyper-prior distribution on the scale parameter of the gene effect size distributions (e.g., t^{RNA+} in Supplementary Materials 1).

(a) Logarithmic Score (lower is better). (b) Brier Score (lower is better).

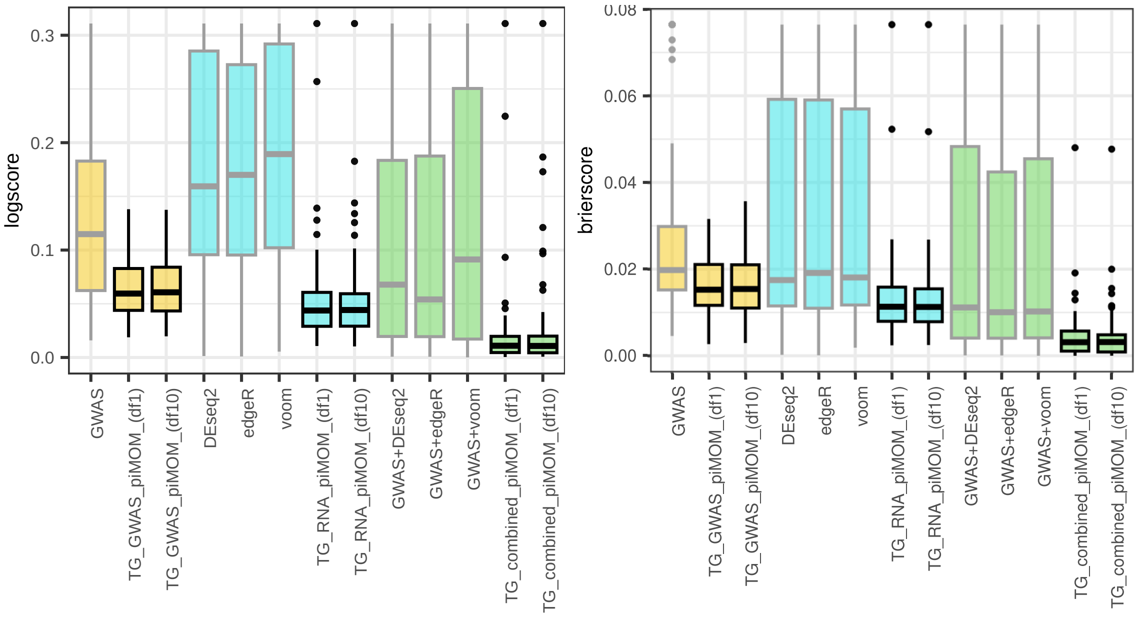

Figure 7. Boxplots of logarithmic scores (a) and Brier scores (b). The last term in the "TG" models indicates the degrees of freedom placed on the hyper-prior for the dispersion parameter of the RNA-seq model.

$N(0, 10^{-3})$) or tuned to the region that is scientifically meaningful (e.g., the half-piMOM excludes very small values and the mass is concentrated away from extremely large odds ratios). An exception to this is the hyperparameter which determines the degrees of freedom of the hyper-prior placed on the precision for the log-Normal prior of the negative binomial dispersion. We performed an additional set of simulations to test the sensitivity to this hyperparameter. The hyper-prior is a half-t distribution with degrees of freedom $\nu = 4$ in all results in the manuscript. Figure 7 displays results of simulation studies with $\nu = 1$ and $\nu = 10$. The results appear to be insensitive to this change in hyper-prior, with MCMC output seeming to differ only up to Monte Carlo error.

5.6 *Genes with effects in only one experiment type*

Our model was designed to identify genes with weak signal in multiple data types through borrowing of strength. The simulation in the manuscript demonstrates that the model performs better than competitors at this task. We think that it is biologically reasonable that some some genes are associated with disease status but have signal in a subset of the included data types (e.g., a gene may exhibit differential expression between the PD and control groups but the minor allele proportions may be the same). We explore whether our model can detect these genes through an additional simulation study. In this set of simulations, data are generated such that some genes are non-null in one experiment type only. Figure 8 displays the results of two simulations with 250 genes where six genes were simulated with effects in both branches and an additional four genes are simulated with an effect only in one branch. The first two rows of the figure demonstrate the sub-models performance which indicates that genes without effects are not identified in these sub-model runs. The joint model (bottom row) indicates two important features of our model; first, that some genes that have signal in one model only are identified in the joint model and, second, that some genes

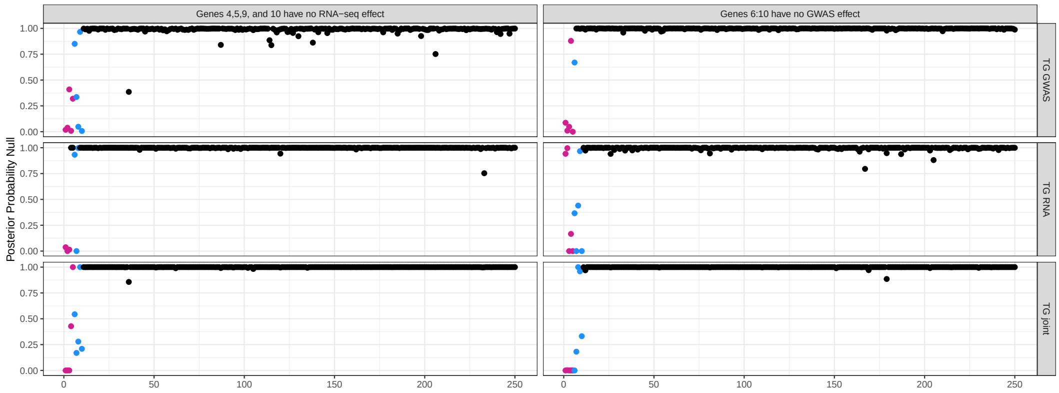

Figure 8. Simulation results with genes that have effect in a single experiment type. Each column demonstrates our model's performance on one simulated set of 250 genes. In the first column, the first 10 genes are generated with an effect in the GWAS branch and six of these first 10 are generated with an effect in the RNA-seq branch. In the second column, six of the first 10 genes are simulated with an effect in the GWAS branch and the first 10 are simulated with an effect in the RNA-seq branch.

that have relatively weak signal in one of the sub-models are identified by the joint model. Boxplots of comparison metrics like in Figure 6 tell the same story as in other simulations; our joint model performs better than p-value combinations of competing models.

5.7 *Missing genes*

Our model seamlessly handles genes which are missing in one of the data types in a standard, fully Bayesian, fashion that treats missing data as parameters to be estimated. Here the missing values are simply drawn from their posterior predictive distributions within the MCMC sampler. This imputation approach means that any signal from genes that are missing in one data type must be captured from the data in a single study. This missingness makes it more challenging for the model to identify these non-null genes.

The challenge of identifying non-null genes which are missing in one data type is not unlike the challenge in identifying genes which are simulated with effects in only one data type, a scenario which was discussed in the previous section. In this section we briefly show that our model is still capable of identifying non-null genes when the data are missing in one experiment. Figure 9 shows the posterior probabilities of inclusion in the null group from three simulations which differ only in the sampling variability in the data generation. In each of these simulations gene number 1 is missing in the RNA-seq dataset (we generated data just as we had for other experiments but replaced this gene's data with NAs). The posterior probability of inclusion in the null group is 1.0 in the RNA-only model. In the first two simulations we see that the small signal that is detectable in the GWAS-only model is enough so that the posterior probability of inclusion in the null group is near 0.5 in the joint model. In the third experiment (column 3) we see that this gene has a strong signal in the GWAS only model and thus it is clearly identified as non-null by the joint model.

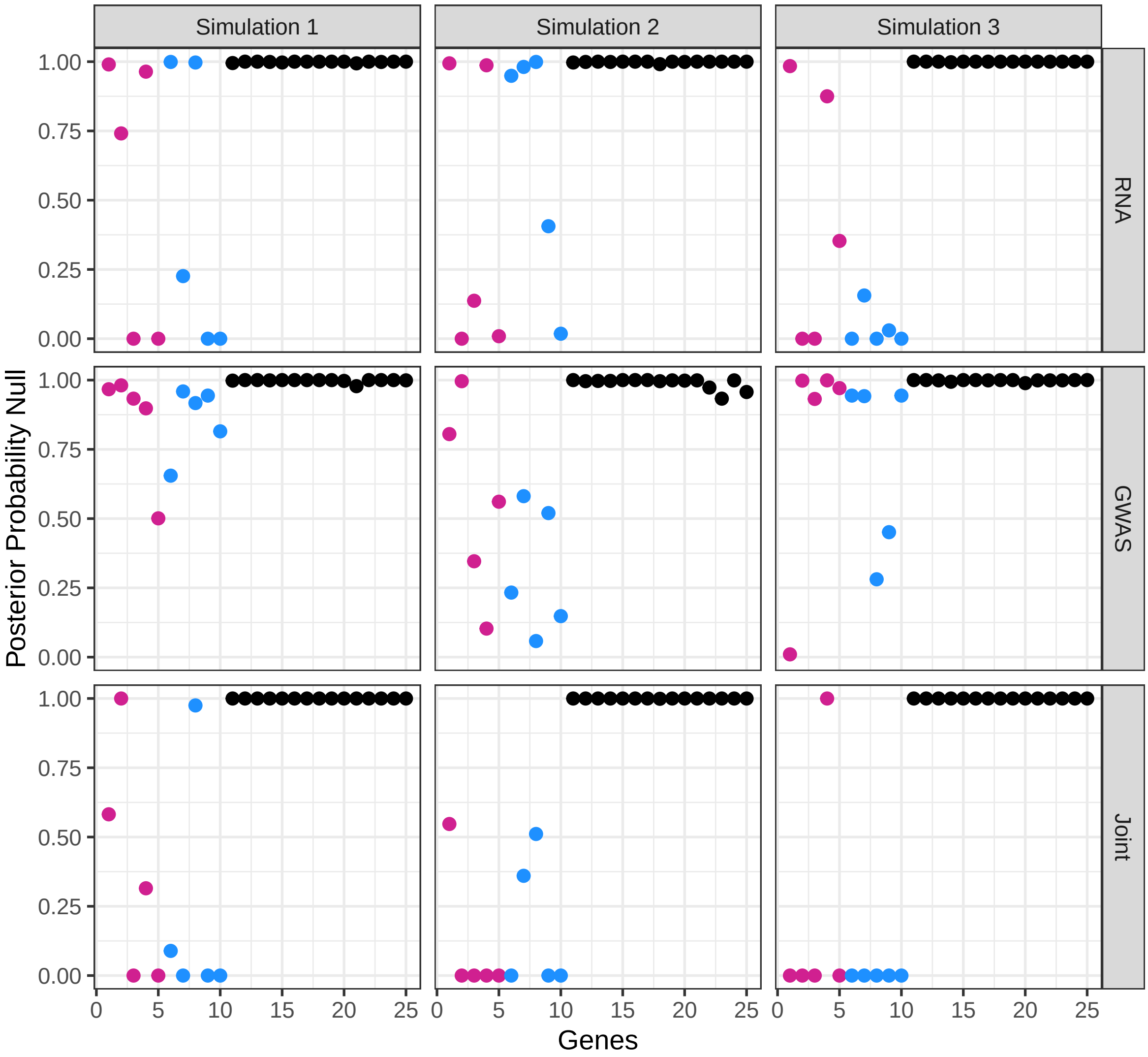

Figure 9. Simulation results with a gene that only has data in one experiment type. Each column demonstrates our model's performance on one simulated set of 100 genes (only 25 are shown for clarity). In all three simulations gene number 1 is missing from the RNA-seq dataset.

5.8 *Simulation Run times*

Computation is always a challenge with MCMC methods. In this section we share boxplots of run times for the simulation in the main text (Figure 10). This figure shows that the RNA only model is quite fast when compared to the GWAS model. We note that the piMOM models are slower than the local models as we had to write custom distributions instead of taking advantage of previous work that speeds up computation with the normal distribution.

5.9 *Sensitivity to Conditional Independence Assumption*

Our model implicitly assumes that RNA counts are independent conditional on PD status. This is more lenient than the full independence assumed in the conventional models used for comparison. It is plausible that both independence

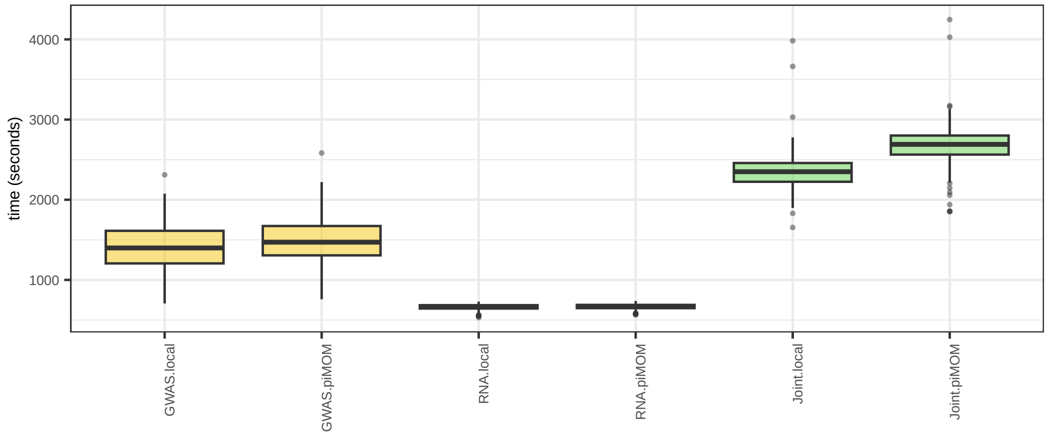

Figure 10. Boxplots which show the computation time (in seconds) of each of our models for each of the 300 simulation in the main text.

assumptions are violated through, for example, differences in the cellular composition of each sample. We assessed our model through simulations with RNA-seq data which are generated by adding signal to a real RNA-seq dataset. We expect these synthetic data to reflect potential violations of our assumptions that may also be present in the real data. In the following, we simulate dependent RNA-seq data to assess the sensitivity of our model to known violations of conditional independence.

The package used for RNA-seq data generation in our simulations (`seqgendiff`) has a method to add surrogate variables. The inclusion of these surrogates imposes dependence across genes. The surrogate variable information is ignored in the analysis of the data. Subfigure 11(a) shows results of one set of simulations in which the RNA-seq data are generated with three surrogates of differing strength (weaker, equal to, and stronger than the PD signal) and prevalence (affecting 15% to 50% of the genes).

A second method for generating data which breaks the conditional independence assumption is to add signal to the RNA-seq data twice; the first signal is a synthetic covariate that will be ignored in the subsequent analysis and the second second signal is the PD signal. This introduces dependence between genes that the model does not account for. To accomplish this we perform the binomial thinning of Gerard (2020) twice. This two-stage approach ensures that the PD signal is added to RNA-seq data which are known to be dependent. We performed simulations using this two-stage data generation with three different levels of effects for the simulated covariate. The lowest level of dependence involves signal that is the same strength as the gene-effects for the genes with differential expression in PD (a coefficient of 1.4 which is a multiplicative effect of 0.48). The other two levels of dependence include coefficients of 5 and 75 (multiplicative effects of 2.32 and 6.23 respectively). Results in Figure 11 demonstrate that with increasing dependence comes decreasing performance (larger log-scores) of all methods that include the RNA-seq data (the GWAS results are identical as those data are the same in all simulations).

The results from these simulations with known violations of assumptions indicate that the performance of all models degrades as the strength of the conditional dependence strengthens. This set of simulations suggests that a moderate level of dependence does not degrade performance dramatically as Subfigures 11(a) and 11(b) are quite

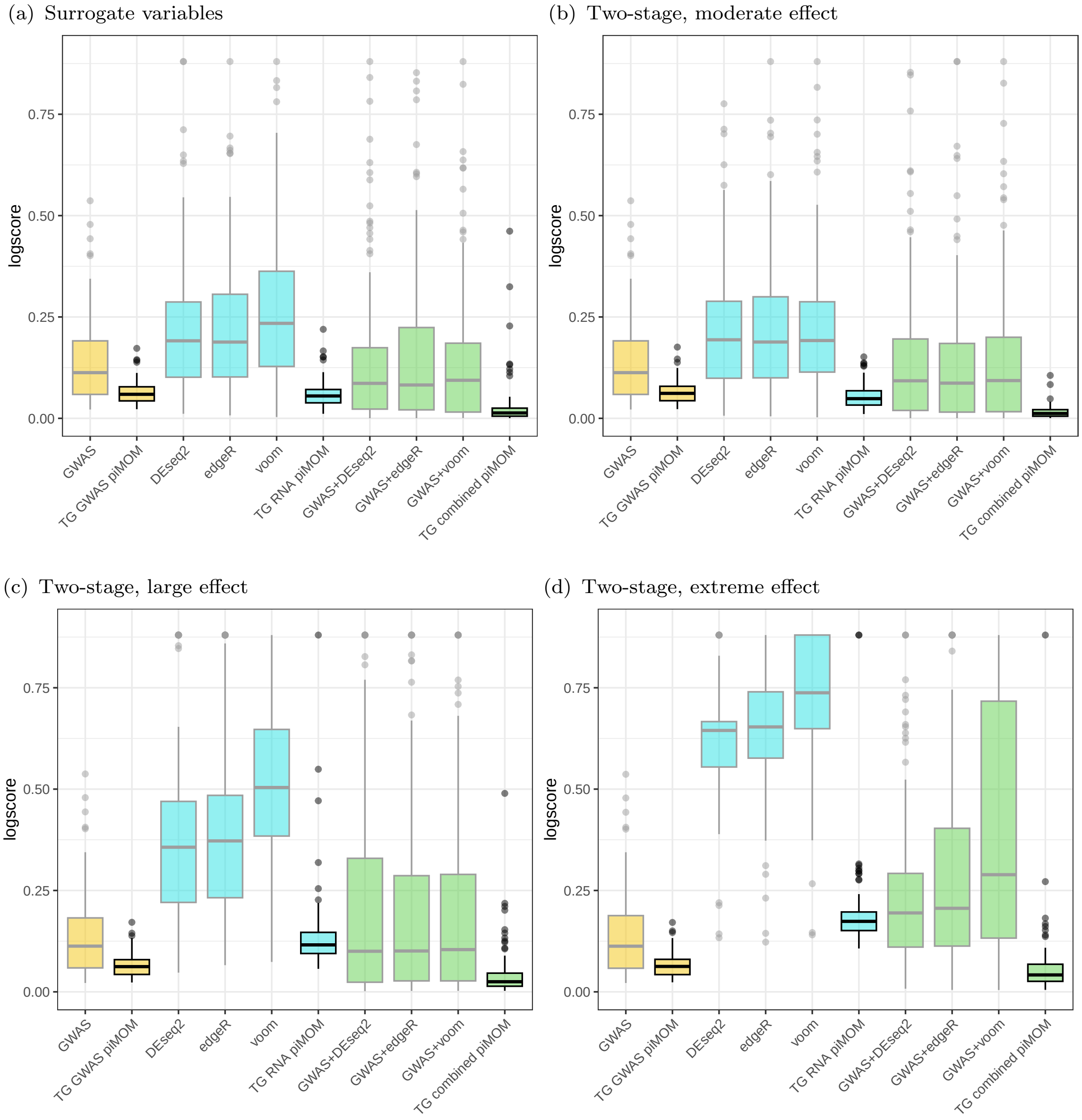

Figure 11. Simulation results when the assumption of conditional independence of RNA counts is broken. RNA-seq data for Subfigure 11(a) were simulated using the surrogate variables feature in `seqgendiff`. Synthetic data for simulations in the other three plots were made by adding spurious signal to the underlying RNA-seq counts before adding the PD signal. The "moderate" effect is the same magnitude as the PD signal.

similar to simulation results without intentionally breaking the conditional independence. Furthermore, our three-groups RNA-seq model appears more robust to violations of conditional independence than conventional models. Finally, we highlight that the increased power of our model, demonstrated in previous simulations, remains across these increasing violations of the assumption of conditional independence.

5.10 *An alternative joint model with summary statistics*

In this section we compare our full joint three-groups model to a version of the model adapted for summary statistics. Our three-groups model analyzes the data from a GWAS and RNA-seq experiment. This is unlike meta-analysis methods which analyze summary statistics computed from these experiments. Summary statistics are convenient because they are often more broadly available than the raw data and of smaller dimensions, so the models that use them as inputs are less computationally demanding. These models may be less powerful than models developed for the raw data.

We adapted our three-groups model for use with summary statistics. We assume that the gene effects estimated from conventional methods (we used GWAS and DEseq2) are approximately Normal random variables that are centered on the true gene effect with standard errors approximated by the respective conventional methods. Let $\hat{\beta}_j^{RNA}$ be the jth estimated gene effect and se_j^{RNA} be the associated standard error as estimated by one of the conventional RNA-seq models. Let $\hat{\beta}_j^{GWAS}$ be the jth estimated gene effect and se_j^{GWAS} be the associated standard error as estimated by one-at-a-time logistic regression. Our summary model is:

$$\begin{aligned}
\hat{\beta}_j^{RNA} &\sim N(\beta_j^{RNA}, \text{se}_j^{RNA}) & \hat{\beta}_j^{GWAS} &\sim N(\beta_j^{GWAS}, \text{se}_j^{GWAS}) \\
\beta_j^{RNA} &\sim \begin{cases} 0 & \text{if } G_j = 1 \\ f^{RNA^+} & \text{if } G_j = 2 \\ f^{RNA^-} & \text{if } G_j = 3 \end{cases} & \beta_j^{GWAS} &\sim \begin{cases} 0 & \text{if } G_j = 1 \\ f^{GWAS^+} & \text{if } G_j = 2 \\ f^{GWAS^-} & \text{if } G_j = 3 \end{cases} \\
f^{RNA^+} &\sim \text{half-piMOM}(t = t^{RNA^+}, r = 2) & f^{GWAS^+} &\sim \text{half-piMOM}(t = t^{GWAS^+}, r = 2) \\
-f^{RNA^-} &\sim \text{half-piMOM}(t = t^{RNA^-}, r = 2) & -f^{GWAS^-} &\sim \text{half-piMOM}(t = t^{GWAS^-}, r = 2) \\
t^{RNA^+} &\sim \text{half-piMOM}(t = 0.05, r = 1) & t^{GWAS^+} &\sim \text{half-piMOM}(t = 0.05, r = 1) \\
t^{RNA^-} &\sim \text{half-piMOM}(t = 0.05, r = 1) & t^{GWAS^-} &\sim \text{half-piMOM}(t = 0.05, r = 1)
\end{aligned}$$

$$G_j \sim \text{Multinomial}\big(n = 1, p_m = (\lambda_1, \lambda_2, \lambda_3)\big)$$
$$(\lambda_1, \lambda_2, \lambda_3) \sim \text{Dirichlet}(1, 1, 1).$$

This summary statistics version of the model is considerably simplified and has very few observations for parameter estimation. The true gene effects β_j^m (where m is either RNA or GWAS) each have a single observation of the estimated gene effect $\hat{\beta}_j^m$. The group membership G_j must be estimated from the two estimated gene effects ($\hat{\beta}_j^{RNA}$ and $\hat{\beta}_j^{GWAS}$) and their associated standard errors. We expect that the summary model would perform better in cases where there are multiple experiments of each type. In addition, an analysis with multiple experiments in each data type would benefit even more from the computational advantages of the summary model.

Figure 12 displays results from two simulation studies that compare our raw-data three-groups model to this summary statistics model. The first study (Subfigure 12(a)) uses the same simulation data that were used to assess our full three-groups model in the manuscript. These results demonstrate that this summary model handily outperforms p-value combinations and is quite competitive with our raw data model. The real data have 34 genes which are only

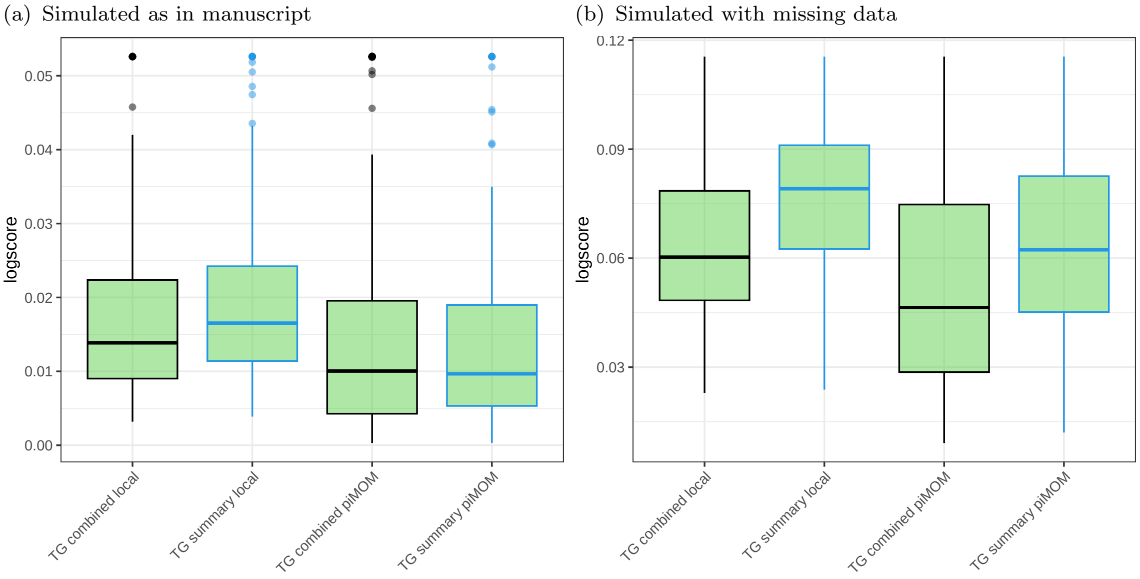

Figure 12. Simulation results comparing our full three-groups model and our three-groups model adapted to summary statistics. Subfigure 12(a) has the same results for our full three-groups model as the main results in the manuscript. Subfigure 12(b) is from simulations where a few genes have been generated with data only in the GWAS branch of the study.

measured in the GWAS experiment. For this reason we also performed a simulation study to assess the summary model with data that were generated with missingness (Subfigure 12(b)). In this second study, a handful of genes were generated with no information in the RNA-seq experiment (as in Section 5.7 of this Supplement). Here we see that the raw data model more clearly outperforms the summary model.

The stark computational advantage of the summary model may be enough of a benefit that it overrides a decrease in power compared to the full model. For this reason, we analyzed the real data using the summary model. Results from this analysis were disappointing; all genes but one (*CHCHD6*) have a posterior probability of inclusion in the null group that is greater than 0.5. Two other genes (*CDIP1* and *DUSP1*) have posterior probability of inclusion in the null group that is between 0.5 and 0.95.

The results of our simulations and real data analysis suggest that a complete investigation into the summary model is needed. The model appears to do well on simulated data but power degrades more rapidly than the raw data model when we move away from perfect simulation scenarios. One area for future exploration has to do with the distinction between estimating gene effects in a joint model versus one-at-a-time models. The gene effects in the GWAS branch of our summary model are estimated using one-at-a-time logistic regression. By contrast, our raw data model jointly estimates gene effects and thus it is capable of borrowing strength across genes. We conjecture that the one-at-a-time GWAS data generation used in our simulations obscures this advantage of the raw data model.

6. An alternative SNV-to-gene mapping

Our GWAS data are collected on the SNV level but our model is on the gene level. This discrepancy requires us to map SNVs to genes. In the manuscript we have reported results using a binary mapping which indicates whether or

not there is a minor allele in each gene. Here we report results of an alternative approach that sums the number of SNVs in or near each coding region. Our model is immediately applicable to this setting when the counts are centered and scaled as part of the preprocessing. When the sum-mapped GWAS data are analyzed with both the GWAS-only TG model and the joint TG model (with half-piMOM hyperpriors on the gene effects) our model finds two non-null genes. The two genes are *CHCHD6* (which was found in the indicator-mapped data) and *NSF* (which was found by both DESeq2 and the joint GWAS-DESeq2 models with the indicator mapping). In the GWAS-only model these genes had empirical posterior inclusion probability of 1.0 and 0.9997 respectively. The next highest inclusion probability was 0.1122. The joint model included both of these interesting genes in all 10000 of the MCMC iterations (post warm-up) and the next largest inclusion probability was 0.0407.

7. Parkinson's Disease Data Analysis

7.1 *Description of the Data*

The data that we used used for the GWAS branch of this study came from the International Parkinson's Disease Genomics Consortium (IPDGC) NeuroX Dataset (Nalls et al., 2014). Due to the intense computational burden of running the full MCMC, we analyzed a subset the full genome. We chose the 2,000 genes that exhibited the largest differential expression between the cortex and the substantia nigra (the region most affected in PD) in Agarwal et al. (2020), as well as 19 additional genes which seemed promising in exploratory analyses. We associated each allele with a particular gene if it was annotated within that gene in the GWAS data (including intronic and 5' or 3' UTR plus 3kbp). This resulted in a list of 53,559 variants associated with the 2,019 genes. The NeuroX dataset included 1,734 of the 2,019 genes on our list, sequenced from 11,402 individuals. We then summarized the SNV data to the gene level using an indicator function for whether a given gene was mapped to at least one SNV. In our analysis, we also included the subject-specific covariates age and sex from the NeuroX dataset, and used PD status as the response for all individuals.

The RNA-seq data that we used came from the Parkinson's Progression Markers Initiative (PPMI), obtained from PPMI upon request. We included individuals who were identified as healthy controls or untreated PD cases, resulting in data for 370 individuals. We extracted RNA fragment counts for each individual for genes that appeared in the same list of 2,019 genes, resulting in data on 1,697 genes (37 genes only had data in the NeuroX dataset). Age, years of education, race, sex, and the phase of the PPMI study are included as covariates.

7.2 *Results*

In the following we classify genes as deleterious or beneficial using the so-called median probability model, hereafter MM, wherein genes are included in a group if the corresponding posterior inclusion probability is greater than 0.5 (Barbieri and Berger, 2004).

The joint three-groups model with local priors identifies three beneficial and four deleterious genes. The three beneficial genes are *CDIP1*, *CHCHD6*, and *CNTNAP2*. These genes are known to be involved in dysregulated

pathways in PD; mitochondrial function (Bose and Beal, 2019; Zaltieri et al., 2015; Moon and Paek, 2015) , synaptic function (Clayton and George, 1998; Morais et al., 2009; Bagetta et al., 2010), and apoptosis (Tatton et al., 2003; Mochizuki et al., 1996; Lev et al., 2003). The four deleterious genes are *DUSP1*, *FAM49B*, *IFRD1*, and *SYTL3* also all have functions in previously implicated PD pathways such as mitochondrial function (Bose and Beal, 2019; Zaltieri et al., 2015; Moon and Paek, 2015), stress responses (Zhao et al., 2010; Chang et al., 2020), autophagy (Liu et al., 2008; Wang et al., 2016), vesicular trafficking and endocytosis (Singh and Muqit, 2020; Perrett et al., 2015; Esposito et al., 2012). Three of these genes (*CDIP1*, *CNTNAP2*, *DUSP1*) have connections with PD in the literature (Li et al., 2022; Brehm et al., 2015; Usenko et al., 2021). The other genes collectively play roles in pathways previously associated with Parkinson's disease, rendering them plausible candidates for further investigation. The estimated effect sizes (conditional on inclusion in the respective non-null groups) are reported in Table 1 of the main manuscript which contains the union of the top 20 genes identified as non-null by the three-groups joint models.

The non-local, joint three-groups model identifies eight genes as beneficial and ten genes as deleterious groups. These 18 genes were in their respective groups for all 10,000 post burn-in MCMC iterations. The two other genes in the top 20 non-null genes were in each of the three groups for at least 10% of the MCMC iterations. Five of the 20 interesting genes are also identified in the local model (*CHCHD6*, *CNTNAP2*, *DUSP1*, *FAM49B*, and *DPP10*) and the 21st most interesting gene in this model (*CDIP1*) was also identified by the local model. Several genes identified by this non-local model are linked to the PD literature. *CNTNAP4*, *CXCR4*, *FCGR2A*, and *PTPRN2* are directly linked to PD in the literature (Hu et al., 2024; Zhang et al., 2020; Ma et al., 2023; Bonham et al., 2018; Gu et al., 2023; Schilder and Raj, 2022; Chuang et al., 2019; Kochmanski et al., 2022, and references therein). *EFCAB6* has been studied in connection with a mutation known to be associated with early-onset PD in Strobbe et al. (2018). Several others can be linked to pathways which have been studied in relationship with PD though we are are unaware of work which directly links these other genes to PD. These pathway links include *ATP8B4*'s association with the innate immune system, *FRAS1*'s link to ERK signaling, and *JARID2*'s interaction with the Polycomb repressive complex 2, all of which have been implicated or have relation to PD in Liu et al. (2021); Tan et al. (2020); Albert-Gascó et al. (2020); Toskas et al. (2022) respectively.

8. MCMC trace plots

Example trace plots from our joint three-groups model with piMOM priors on gene effects are given in Figures 13 and 14. Included are trace plots for parameters from the six genes with the lowest posterior probability of inclusion in the null group and one gene that is in null in all mcmc samples. Trace plots for covariates and other hyper-parameters are also included.

9. Volcano Plots

It is common to use volcano plots to identify the most promising genes in RNA-seq analyses. These plots conventionally have the log fold change on the horizontal axis and the negative log p-value on the vertical axis which allows

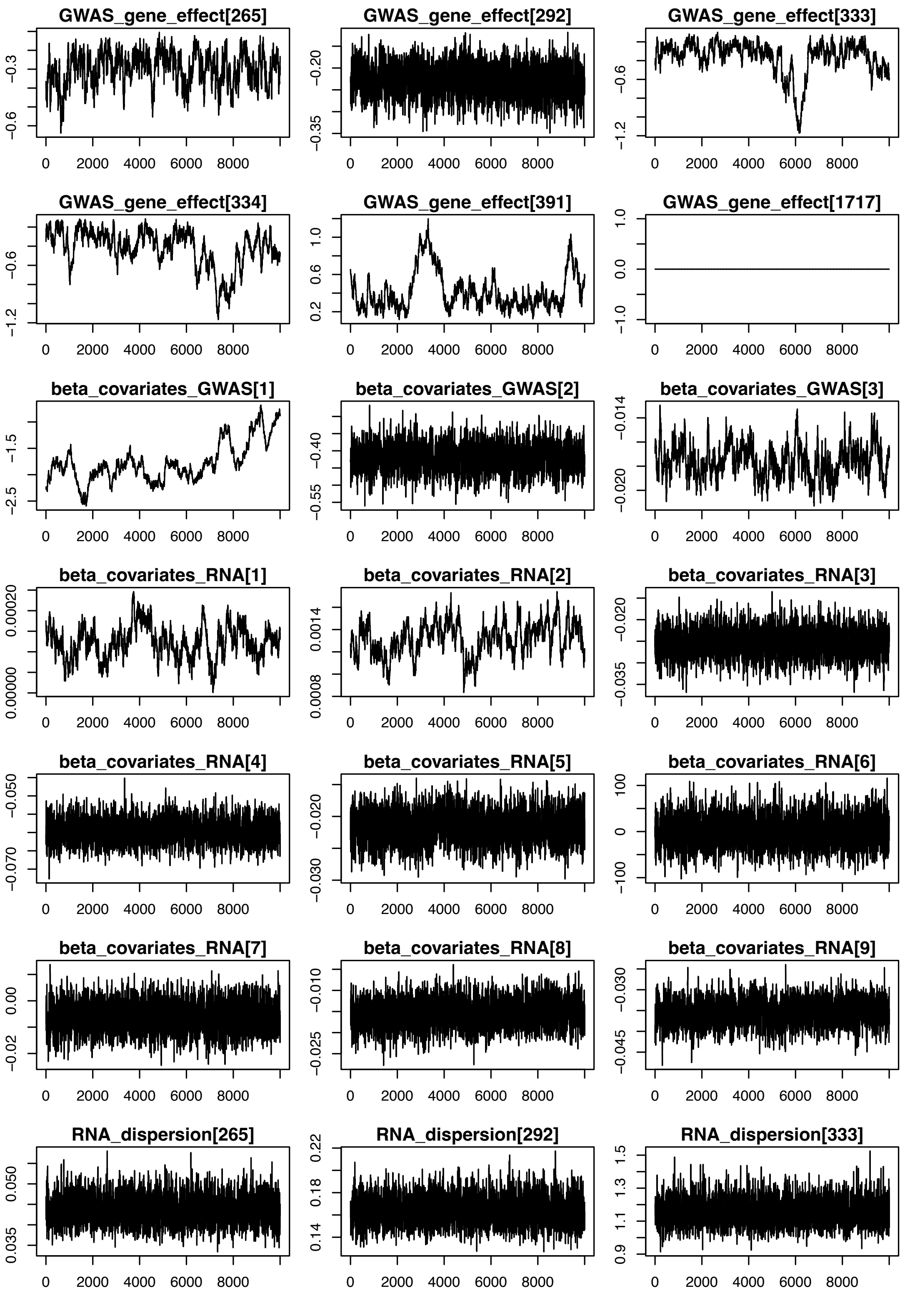

Figure 13. Coda plots 1

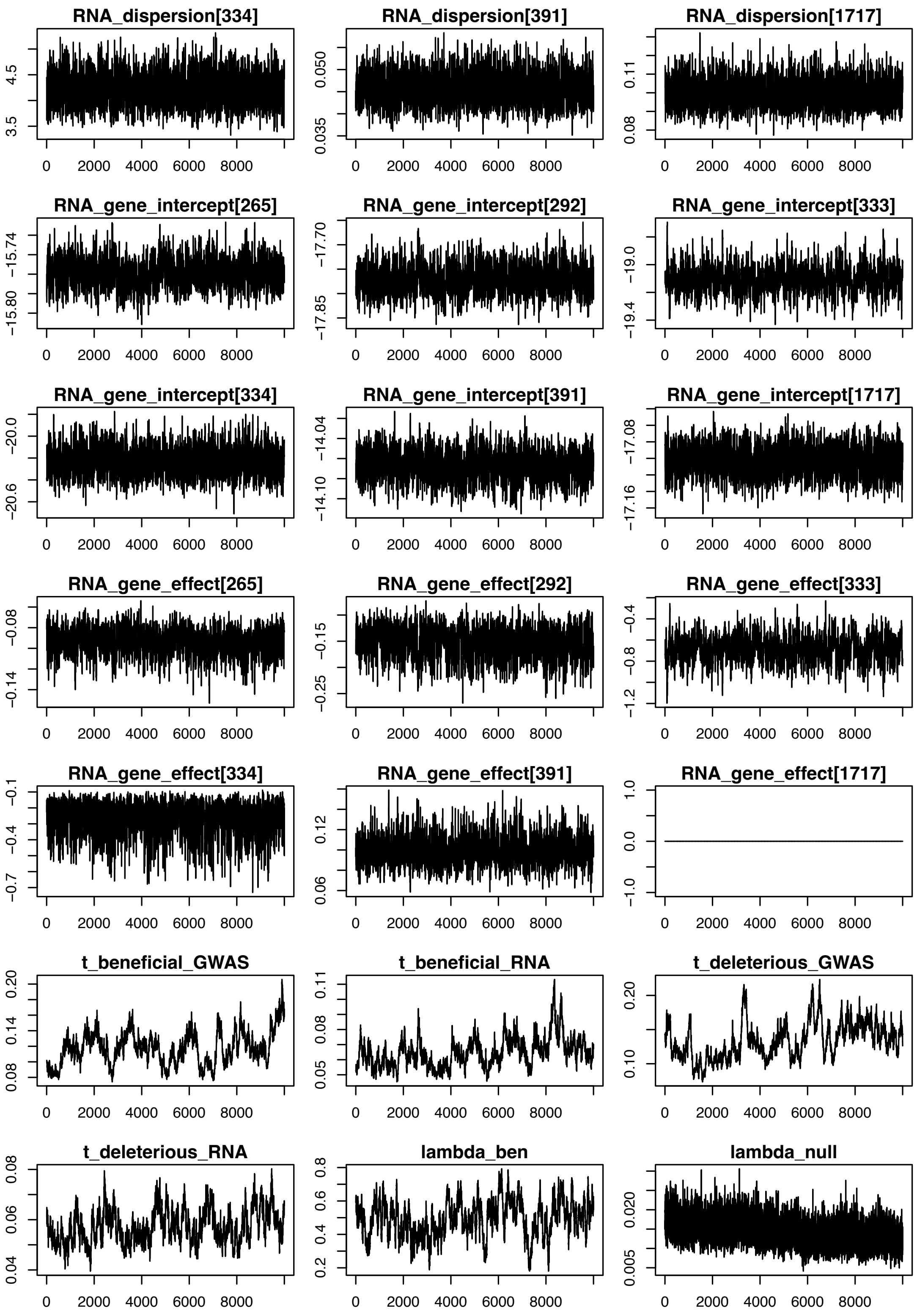

Figure 14. Coda plots 2

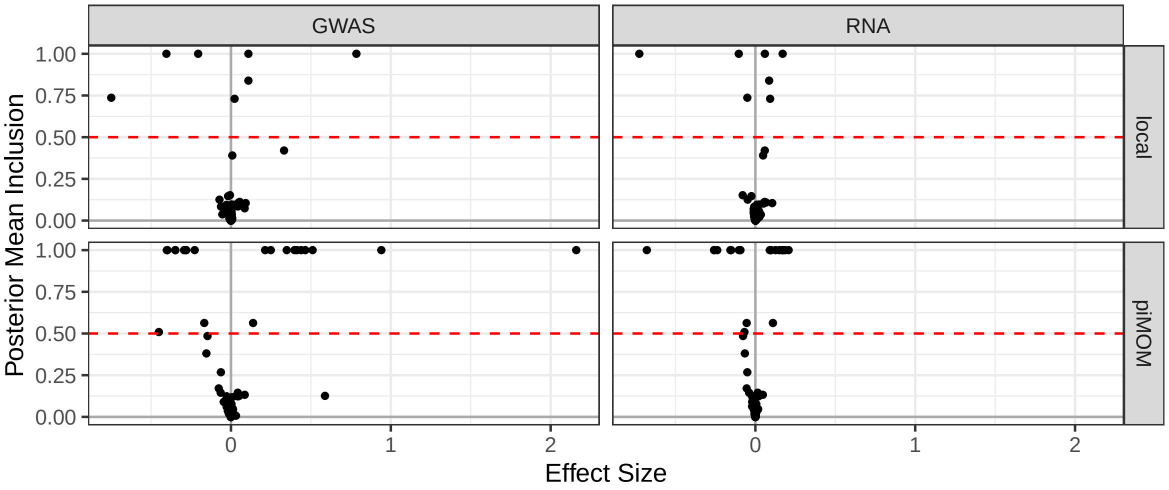

Figure 15. Volcano plots for the local model (top row) and piMOM model (bottom row). Effect sizes are the marginal log odds ratio (GWAS) and log fold change (RNA). The red dashed line indicates the MM cutoff.

investigators to quickly subset genes which are both statistically significant and have large effect sizes. Our model does not benefit from these plots in the same manner due to the inherent sparsity. Nevertheless, we have created a version of these plots in Figure 15 with the marginal log effect size on the horizontal axis and posterior mean of inclusion on the vertical axis. The vast majority of genes are not visible because they are piled up exactly at the origin. These plots highlight the non-local nature of the piMOM model; no included genes have small effect sizes.

10. Non-null genes from all models

Table 2 displays a list of all of the genes that can be considered non-null by any of the models we ran on the real data. Any gene with a posterior inclusion probability in the null group that was less than 0.5 is considered a non-null gene. This median model (MM) cutoff is applied to our three-groups models except the local GWAS model (even with a cutoff of 0.0001 there were 94 non-null genes). Genes are considered non-null in the conventional models if their lFDR value is below 0.05. Once again, this cutoff is different because the MM cutoff finds too many non-null genes to be useful (the MM for GWAS combined with `edgeR`, `limma+voom`, and `DESeq2` results in the inclusion of 418, 220, and 533 genes respectively whereas the 0.05 cutoff results in 14, 8, and 27 non-null genes respectively).

	Gene	TG RNA local	TG RNA piMOM	TG GWAS local	TG GWAS piMOM	TG joint local	TG joint piMOM	`edgeR`	`voom`	`DESeq2`	GWAS	GWAS+`edgeR`	GWAS+`voom`	GWAS+`DESeq2`
1	ACTR10			*										
2	ADAMTS19			*										
3	AFF3									*				
4	ALOX5AP			*						*				
5	ANK3			*										
6	ANKFN1				*									

Continued on next page

	Gene	TG RNA local	TG RNA piMOM	TG GWAS local	TG GWAS piMOM	TG joint local	TG joint piMOM	`edgeR`	`voom`	`DESeq2`	GWAS	GWAS+`edgeR`	GWAS+`voom`	GWAS+`DESeq2`
7	AOAH			*										
8	APOD	*						*				*		
9	ARF1			*										
10	ARHGEF26			*										
11	ATP2B1			*										
12	ATP2C1			*										
13	ATP5A1			*										
14	ATP6V0B								*	*				
15	ATP8B4						*							
16	AZI2				*									
17	BDH1			*										
18	C10orf90						*							
19	C2CD5			*										
20	C6orf136			*										
21	CAMLG			*										
22	CAMTA1				*									
23	CANX			*										
24	CCDC136			*										
25	CCPG1			*						*				
26	CD180									*				
27	CD200	*	*					*						
28	CD82						*							
29	CD83	*						*				*		
30	CDH7			*										
31	CDIP1				*	*	*				*	*	*	*
32	CDK14									*				
33	CHCHD3			*										
34	CHCHD6			*	*	*	*				*	*	*	*
35	CHD6			*										
36	CLEC7A									*		*	*	*
37	CNTNAP2	*	*			*	*	*				*		
38	CNTNAP4						*							
39	CNTNAP5			*	*									
40	CREM									*				
41	CSMD1				*									
42	CTSB			*										
43	CXCR4						*							
44	CYB5R1			*										
45	DACH1				*									
46	DDIT3			*										
47	DLGAP1						*							
48	DOCK4							*		*				
49	DPP10						*							
50	DPYSL5			*										
51	DUSP1	*		*		*	*	*	*	*		*	*	*
52	EDIL3				*									
53	EFCAB6						*							
54	EFEMP1			*										
55	ENPP2			*										
56	EVL			*										
57	FAM49B					*	*			*				
58	FAM98A			*										
59	FBXL17			*										
60	FCGR2A						*			*				*
61	FGD4	*						*	*	*		*	*	*
62	FIGN						*							
63	FILIP1L							*						
64	FMNL3			*										*
65	FNDC3A			*										
66	FOCAD				*									
67	FOS							*	*	*		*	*	*
68	FRAS1			*			*							
69	FSD1			*										
70	FSTL5				*									

Continued on next page

	Gene	TG RNA local	TG RNA piMOM	TG GWAS local	TG GWAS piMOM	TG joint local	TG joint piMOM	`edgeR`	`voom`	`DESeq2`	GWAS	GWAS+`edgeR`	GWAS+`voom`	GWAS+`DESeq2`
71	GABARAPL1	*						*	*	*			*	*
72	GABRG3			*										
73	GALNT13				*									
74	GLIS3				*									
75	GNB4									*				
76	GPR183			*										
77	GRIA2			*										
78	HAGH			*										
79	HAP1			*										
80	HDAC9				*									
81	HERPUD1									*				
82	HIF1A									*				
83	HLA-DPA1			*										
84	HSPA2			*										
85	HSPA6				*						*	*	*	*
86	HSPD1													*
87	IDH3A			*										
88	IFIT3							*		*				*
89	IFRD1	*				*		*	*	*				*
90	IQCA1			*										
91	ITM2B									*				
92	JARID2						*							
93	JPH4			*										
94	KATNB1			*										
95	KBTBD8			*										
96	KCNJ6			*										
97	KHDC1			*										
98	KHDRBS3			*										
99	KIAA1958											*		*
100	KLF6									*				
101	KLK6			*										
102	LAPTM5			*										
103	LIN7A									*				*
104	LINC01197			*										
105	LPAR6			*										
106	LPCAT2									*				
107	LRFN5						*							
108	LRRK2									*				
109	LRRN1			*										
110	LYRM1									*				*
111	MAP2K4									*				
112	MAP4K5			*										
113	MARCKSL1			*										
114	MAST2				*									
115	MCTP1							*		*				*
116	MFSD4A			*										
117	MOCS2			*										
118	MRPL55			*										
119	MXD1									*				
120	NCALD			*										
121	NKAIN2			*										
122	NPTN									*				*
123	NSF			*										
124	NUTF2			*										
125	OR2W3									*				
126	ORMDL1			*										
127	PACSIN1			*										
128	PARP8									*				*
129	PCSK5				*									
130	PDK2									*				
131	PDK4													*
132	PELI1	*						*	*	*				*
133	PGAM1			*										
134	PLEKHB1			*										

Continued on next page

	Gene	TG RNA local	TG RNA piMOM	TG GWAS local	TG GWAS piMOM	TG joint local	TG joint piMOM	edgeR	voom	DESeq2	GWAS	GWAS+edgeR	GWAS+voom	GWAS+DESeq2
135	PLEKHB2			*										
136	PLXDC2									*				
137	PTPRD				*									
138	PTPRG				*									
139	PTPRN2						*							
140	PTPRR			*										
141	RAMP1			*										
142	RANBP2			*										
143	RAP1GAP	*	*					*		*		*		*
144	RGS2									*				
145	RHOQ			*										
146	RIN3						*							
147	RNF13									*				
148	RSRP1									*				*
149	S100B			*										
150	SCN1A			*										
151	SCRN2			*										
152	SEMA3B			*										
153	SERINC3			*										
154	SKAP2									*				
155	SLC16A4							*						
156	SLC24A2				*									
157	SLC25A29			*										
158	SLC31A2									*				
159	STRBP							*		*				
160	SYT13			*										
161	SYT5			*										
162	SYTL3					*		*		*		*		*
163	TIMM22			*										
164	TLR6			*						*				*
165	TM2D3									*				
166	TMCC3									*				*
167	TMEM125			*										
168	TMEM181			*										
169	TMEM246			*										
170	TMTC1				*									
171	TMX4									*				
172	TPI1			*										
173	TPST1							*		*		*		*
174	TRANK1									*				
175	TRPS1			*										
176	TUFM			*										
177	TYROBP									*				
178	UQCR10			*										
179	UQCRC1			*										
180	VRK2						*							
181	WDR12			*						*				
182	XYLT1				*									
183	ZDHHC20			*										

Table 2: Genes identified as non-null in at least one of model. A gene is classified as non-null in all models with an "*". The three groups (TG) models use the median model (cutoff at $P_{null} < 0.5$) except for the local GWAS model which included too many genes. We used a cutoff of 0.0001 for this GWAS model which still included 94 genes. All conventional models used a cutoff of 0.05.

11. Non-null genes and effect sizes from TG RNA only and GWAS only models.

	Gene	P_{null} RNA local	P_{ben} RNA local	P_{del} RNA local	P_{null} RNA piMOM	P_{ben} RNA piMOM	P_{del} RNA piMOM	P_{null} GWAS local	P_{ben} GWAS local	P_{del} GWAS local	P_{null} GWAS piMOM	P_{ben} GWAS piMOM	P_{del} GWAS piMOM	RNA effect local	Dispersion local	RNA effect piMOM	Dispersion piMOM	GWAS effect local	GWAS effect piMOM
1	ACTR10	1.00	0.00	0.00	1.00	0.00	0.00	0.00	0.00	1.00	1.00	0.00	0.00	x	x	x	x	179.34	x
2	ADAMTS19	1.00	0.00	0.00	1.00	0.00	0.00	0.00	0.00	1.00	1.00	0.00	0.00	x	x	x	x	2.37	x
3	ALOX5AP	1.00	0.00	0.00	1.00	0.00	0.00	0.00	1.00	0.00	1.00	0.00	0.00	x	x	x	x	0.03	x
4	ANK3	1.00	0.00	0.00	1.00	0.00	0.00	0.00	0.00	1.00	1.00	0.00	0.00	x	x	x	x	3.36	x
5	ANKFN1	1.00	0.00	0.00	1.00	0.00	0.00	1.00	0.00	0.00	0.00	1.00	0.00	x	x	x	x	x	0.71
6	AOAH	1.00	0.00	0.00	1.00	0.00	0.00	0.00	0.00	1.00	1.00	0.00	0.00	x	x	x	x	2.92	x
7	APOD	0.22	0.10	0.68	1.00	0.00	0.00	1.00	0.00	0.00	1.00	0.00	0.00	0.45	5.16	x	x	x	x
8	ARF1	1.00	0.00	0.00	1.00	0.00	0.00	0.00	0.00	1.00	1.00	0.00	0.00	x	x	x	x	20.39	x
9	ARHGEF26	1.00	0.00	0.00	1.00	0.00	0.00	0.00	0.00	1.00	1.00	0.00	0.00	x	x	x	x	1.95	x
10	ATP2B1	1.00	0.00	0.00	1.00	0.00	0.00	0.00	1.00	0.00	1.00	0.00	0.00	x	x	x	x	2.04	x
11	ATP2C1	1.00	0.00	0.00	1.00	0.00	0.00	0.00	1.00	0.00	1.00	0.00	0.00	x	x	x	x	2.43	x
12	ATP5A1	1.00	0.00	0.00	1.00	0.00	0.00	0.00	1.00	0.00	1.00	0.00	0.00	x	x	x	x	0.16	x
13	AZI2	1.00	0.00	0.00	1.00	0.00	0.00	1.00	0.00	0.00	0.00	0.00	1.00	x	x	x	x	x	1.82
14	BDH1	1.00	0.00	0.00	1.00	0.00	0.00	0.00	1.00	0.00	1.00	0.00	0.00	x	x	x	x	0.46	x
15	C2CD5	1.00	0.00	0.00	1.00	0.00	0.00	0.00	1.00	0.00	1.00	0.00	0.00	x	x	x	x	0.08	x
16	C6orf136	1.00	0.00	0.00	1.00	0.00	0.00	0.00	1.00	0.00	1.00	0.00	0.00	x	x	x	x	3.03	x
17	CAMLG	1.00	0.00	0.00	1.00	0.00	0.00	0.00	0.00	1.00	1.00	0.00	0.00	x	x	x	x	0.28	x
18	CAMTA1	1.00	0.00	0.00	1.00	0.00	0.00	1.00	0.00	0.00	0.00	0.00	1.00	x	x	x	x	x	0.41
19	CANX	1.00	0.00	0.00	1.00	0.00	0.00	0.00	0.00	1.00	1.00	0.00	0.00	x	x	x	x	0.03	x
20	CCDC136	1.00	0.00	0.00	1.00	0.00	0.00	0.00	1.00	0.00	1.00	0.00	0.00	x	x	x	x	0.25	x
21	CCPG1	1.00	0.00	0.00	1.00	0.00	0.00	0.00	0.00	1.00	1.00	0.00	0.00	x	x	x	x	2.27	x
22	CD200	0.42	0.46	0.12	0.44	0.49	0.07	1.00	0.00	0.00	1.00	0.00	0.00	0.77	0.43	0.75	0.43	x	x
23	CD83	0.37	0.51	0.12	1.00	0.00	0.00	1.00	0.00	0.00	1.00	0.00	0.00	0.84	0.16	x	x	x	x
24	CDH7	1.00	0.00	0.00	1.00	0.00	0.00	0.00	0.00	1.00	1.00	0.00	0.00	x	x	x	x	5.89	x
25	CDIP1	1.00	0.00	0.00	1.00	0.00	0.00	1.00	0.00	0.00	0.00	1.00	0.00	x	x	x	x	x	0.49
26	CHCHD3	1.00	0.00	0.00	1.00	0.00	0.00	0.00	0.00	1.00	1.00	0.00	0.00	x	x	x	x	2.26	x
27	CHCHD6	1.00	0.00	0.00	1.00	0.00	0.00	0.00	0.00	1.00	0.00	1.00	0.00	x	x	x	x	0.41	0.8
28	CHD6	1.00	0.00	0.00	1.00	0.00	0.00	0.00	0.00	1.00	1.00	0.00	0.00	x	x	x	x	2.71	x
29	CNTNAP2	0.22	0.10	0.68	0.00	1.00	0.00	1.00	0.00	0.00	1.00	0.00	0.00	0.48	1.17	0.5	1.16	x	x
30	CNTNAP5	1.00	0.00	0.00	1.00	0.00	0.00	0.00	1.00	0.00	0.39	0.14	0.47	x	x	x	x	5.58	0.61
31	CSMD1	1.00	0.00	0.00	1.00	0.00	0.00	1.00	0.00	0.00	0.00	1.00	0.00	x	x	x	x	x	1.64

Continued on next page

	Gene	P_{null} RNA local	P_{ben} RNA local	P_{del} RNA local	P_{null} RNA piMOM	P_{ben} RNA piMOM	P_{del} RNA piMOM	P_{null} GWAS local	P_{ben} GWAS local	P_{del} GWAS local	P_{null} GWAS piMOM	P_{ben} GWAS piMOM	P_{del} GWAS piMOM	RNA effect local	Dispersion local	RNA effect piMOM	Dispersion piMOM	GWAS effect local	GWAS effect piMOM
32	CTSB	1.00	0.00	0.00	1.00	0.00	0.00	0.00	0.00	1.00	1.00	0.00	0.00	x	x	x	x	5.36	x
33	CYB5R1	1.00	0.00	0.00	1.00	0.00	0.00	0.00	1.00	0.00	1.00	0.00	0.00	x	x	x	x	5.62	x
34	DACH1	1.00	0.00	0.00	1.00	0.00	0.00	1.00	0.00	0.00	0.00	1.00	0.00	x	x	x	x	x	1.53
35	DDIT3	1.00	0.00	0.00	1.00	0.00	0.00	0.00	0.00	1.00	1.00	0.00	0.00	x	x	x	x	0.1	x
36	DPYSL5	1.00	0.00	0.00	1.00	0.00	0.00	0.00	0.00	1.00	1.00	0.00	0.00	x	x	x	x	2.77	x
37	DUSP1	0.22	0.10	0.68	1.00	0.00	0.00	0.00	0.00	1.00	1.00	0.00	0.00	1.18	0.09	x	x	6.12	x
38	EDIL3	1.00	0.00	0.00	1.00	0.00	0.00	1.00	0.00	0.00	0.43	0.47	0.10	x	x	x	x	x	0.69
39	EFEMP1	1.00	0.00	0.00	1.00	0.00	0.00	0.00	0.00	1.00	1.00	0.00	0.00	x	x	x	x	2.09	x
40	ENPP2	1.00	0.00	0.00	1.00	0.00	0.00	0.00	1.00	0.00	1.00	0.00	0.00	x	x	x	x	17.02	x
41	EVL	1.00	0.00	0.00	1.00	0.00	0.00	0.00	0.00	1.00	1.00	0.00	0.00	x	x	x	x	0.17	x
42	FAM98A	1.00	0.00	0.00	1.00	0.00	0.00	0.00	0.00	1.00	1.00	0.00	0.00	x	x	x	x	3.85	x
43	FBXL17	1.00	0.00	0.00	1.00	0.00	0.00	0.00	0.00	1.00	1.00	0.00	0.00	x	x	x	x	5.43	x
44	FGD4	0.00	1.00	0.00	1.00	0.00	0.00	1.00	0.00	0.00	1.00	0.00	0.00	1.13	0.06	x	x	x	x
45	FMNL3	1.00	0.00	0.00	1.00	0.00	0.00	0.00	0.00	1.00	1.00	0.00	0.00	x	x	x	x	0.06	x
46	FNDC3A	1.00	0.00	0.00	1.00	0.00	0.00	0.00	1.00	0.00	1.00	0.00	0.00	x	x	x	x	5.18	x
47	FOCAD	1.00	0.00	0.00	1.00	0.00	0.00	1.00	0.00	0.00	0.00	1.00	0.00	x	x	x	x	x	0.7
48	FRAS1	1.00	0.00	0.00	1.00	0.00	0.00	0.00	0.00	1.00	1.00	0.00	0.00	x	x	x	x	4.4	x
49	FSD1	1.00	0.00	0.00	1.00	0.00	0.00	0.00	0.00	1.00	1.00	0.00	0.00	x	x	x	x	2.13	x
50	FSTL5	1.00	0.00	0.00	1.00	0.00	0.00	1.00	0.00	0.00	0.00	1.00	0.00	x	x	x	x	x	0.71
51	GABARAPL1	0.49	0.38	0.13	1.00	0.00	0.00	1.00	0.00	0.00	1.00	0.00	0.00	1.11	0.04	x	x	x	x
52	GABRG3	1.00	0.00	0.00	1.00	0.00	0.00	0.00	0.00	1.00	1.00	0.00	0.00	x	x	x	x	4.58	x
53	GALNT13	1.00	0.00	0.00	1.00	0.00	0.00	1.00	0.00	0.00	0.00	1.00	0.00	x	x	x	x	x	0.7
54	GLIS3	1.00	0.00	0.00	1.00	0.00	0.00	1.00	0.00	0.00	0.00	1.00	0.00	x	x	x	x	x	1.53
55	GPR183	1.00	0.00	0.00	1.00	0.00	0.00	0.00	1.00	0.00	1.00	0.00	0.00	x	x	x	x	0.02	x
56	GRIA2	1.00	0.00	0.00	1.00	0.00	0.00	0.00	0.00	1.00	1.00	0.00	0.00	x	x	x	x	2.08	x
57	HAGH	1.00	0.00	0.00	1.00	0.00	0.00	0.00	0.00	1.00	1.00	0.00	0.00	x	x	x	x	0.15	x
58	HAP1	1.00	0.00	0.00	1.00	0.00	0.00	0.00	1.00	0.00	1.00	0.00	0.00	x	x	x	x	4.58	x
59	HDAC9	1.00	0.00	0.00	1.00	0.00	0.00	1.00	0.00	0.00	0.14	0.81	0.05	x	x	x	x	x	0.74
60	HLA-DPA1	1.00	0.00	0.00	1.00	0.00	0.00	0.00	0.00	1.00	1.00	0.00	0.00	x	x	x	x	4.21	x
61	HSPA2	1.00	0.00	0.00	1.00	0.00	0.00	0.00	0.00	1.00	1.00	0.00	0.00	x	x	x	x	33.82	x
62	HSPA6	1.00	0.00	0.00	1.00	0.00	0.00	1.00	0.00	0.00	0.00	0.00	1.00	x	x	x	x	x	0.6
63	IDH3A	1.00	0.00	0.00	1.00	0.00	0.00	0.00	0.00	1.00	1.00	0.00	0.00	x	x	x	x	0.22	x
64	IFRD1	0.00	1.00	0.00	1.00	0.00	0.00	1.00	0.00	0.00	1.00	0.00	0.00	1.13	0.07	x	x	x	x

Continued on next page

	Gene	P_{null} RNA local	P_{ben} RNA local	P_{del} RNA local	P_{null} RNA piMOM	P_{ben} RNA piMOM	P_{del} RNA piMOM	P_{null} GWAS local	P_{ben} GWAS local	P_{del} GWAS local	P_{null} GWAS piMOM	P_{ben} GWAS piMOM	P_{del} GWAS piMOM	RNA effect local	Dispersion local	RNA effect piMOM	Dispersion piMOM	GWAS effect local	GWAS effect piMOM
65	IQCA1	1.00	0.00	0.00	1.00	0.00	0.00	0.00	0.00	1.00	1.00	0.00	0.00	x	x	x	x	2.34	x
66	JPH4	1.00	0.00	0.00	1.00	0.00	0.00	0.00	0.00	1.00	1.00	0.00	0.00	x	x	x	x	0.01	x
67	KATNB1	1.00	0.00	0.00	1.00	0.00	0.00	0.00	0.00	1.00	1.00	0.00	0.00	x	x	x	x	3.68	x
68	KBTBD8	1.00	0.00	0.00	1.00	0.00	0.00	0.00	0.00	1.00	1.00	0.00	0.00	x	x	x	x	1.59	x
69	KCNJ6	1.00	0.00	0.00	1.00	0.00	0.00	0.00	0.00	1.00	1.00	0.00	0.00	x	x	x	x	3.6	x
70	KHDC1	1.00	0.00	0.00	1.00	0.00	0.00	0.00	0.00	1.00	1.00	0.00	0.00	x	x	x	x	6.61	x
71	KHDRBS3	1.00	0.00	0.00	1.00	0.00	0.00	0.00	0.00	1.00	1.00	0.00	0.00	x	x	x	x	3.33	x
72	KLK6	1.00	0.00	0.00	1.00	0.00	0.00	0.00	0.00	1.00	1.00	0.00	0.00	x	x	x	x	44.74	x
73	LAPTM5	1.00	0.00	0.00	1.00	0.00	0.00	0.00	1.00	0.00	1.00	0.00	0.00	x	x	x	x	2.14	x
74	LINC01197	1.00	0.00	0.00	1.00	0.00	0.00	0.00	0.00	1.00	1.00	0.00	0.00	x	x	x	x	0.45	x
75	LPAR6	1.00	0.00	0.00	1.00	0.00	0.00	0.00	1.00	0.00	1.00	0.00	0.00	x	x	x	x	0.13	x
76	LRRN1	1.00	0.00	0.00	1.00	0.00	0.00	0.00	0.00	1.00	1.00	0.00	0.00	x	x	x	x	3.8	x
77	MAP4K5	1.00	0.00	0.00	1.00	0.00	0.00	0.00	0.00	1.00	1.00	0.00	0.00	x	x	x	x	1.7	x
78	MARCKSL1	1.00	0.00	0.00	1.00	0.00	0.00	0.00	0.00	1.00	1.00	0.00	0.00	x	x	x	x	0.04	x
79	MAST2	1.00	0.00	0.00	1.00	0.00	0.00	1.00	0.00	0.00	0.00	0.00	1.00	x	x	x	x	x	0.68
80	MFSD4A	1.00	0.00	0.00	1.00	0.00	0.00	0.00	1.00	0.00	1.00	0.00	0.00	x	x	x	x	0.25	x
81	MOCS2	1.00	0.00	0.00	1.00	0.00	0.00	0.00	0.00	1.00	1.00	0.00	0.00	x	x	x	x	2.6	x
82	MRPL55	1.00	0.00	0.00	1.00	0.00	0.00	0.00	0.00	1.00	1.00	0.00	0.00	x	x	x	x	25.93	x
83	NCALD	1.00	0.00	0.00	1.00	0.00	0.00	0.00	0.00	1.00	1.00	0.00	0.00	x	x	x	x	0.42	x
84	NKAIN2	1.00	0.00	0.00	1.00	0.00	0.00	0.00	0.00	1.00	1.00	0.00	0.00	x	x	x	x	6.08	x
85	NSF	1.00	0.00	0.00	1.00	0.00	0.00	0.00	1.00	0.00	1.00	0.00	0.00	x	x	x	x	0.48	x
86	NUTF2	1.00	0.00	0.00	1.00	0.00	0.00	0.00	1.00	0.00	1.00	0.00	0.00	x	x	x	x	4.29	x
87	ORMDL1	1.00	0.00	0.00	1.00	0.00	0.00	0.00	1.00	0.00	1.00	0.00	0.00	x	x	x	x	55.44	x
88	PACSIN1	1.00	0.00	0.00	1.00	0.00	0.00	0.00	0.00	1.00	1.00	0.00	0.00	x	x	x	x	3.01	x
89	PCSK5	1.00	0.00	0.00	1.00	0.00	0.00	1.00	0.00	0.00	0.00	1.00	0.00	x	x	x	x	x	2.22
90	PELI1	0.12	0.83	0.05	1.00	0.00	0.00	1.00	0.00	0.00	1.00	0.00	0.00	1.11	0.05	x	x	x	x
91	PGAM1	1.00	0.00	0.00	1.00	0.00	0.00	0.00	0.00	1.00	1.00	0.00	0.00	x	x	x	x	68.49	x
92	PLEKHB1	1.00	0.00	0.00	1.00	0.00	0.00	0.00	1.00	0.00	1.00	0.00	0.00	x	x	x	x	2.32	x
93	PLEKHB2	1.00	0.00	0.00	1.00	0.00	0.00	0.00	0.00	1.00	1.00	0.00	0.00	x	x	x	x	30.29	x
94	PTPRD	1.00	0.00	0.00	1.00	0.00	0.00	1.00	0.00	0.00	0.00	1.00	0.00	x	x	x	x	x	1.49
95	PTPRG	1.00	0.00	0.00	1.00	0.00	0.00	1.00	0.00	0.00	0.00	0.00	1.00	x	x	x	x	x	2.18
96	PTPRR	1.00	0.00	0.00	1.00	0.00	0.00	0.00	0.00	1.00	1.00	0.00	0.00	x	x	x	x	1.56	x
97	RAMP1	1.00	0.00	0.00	1.00	0.00	0.00	0.00	1.00	0.00	1.00	0.00	0.00	x	x	x	x	35.86	x

Continued on next page

	Gene	P_{null} RNA local	P_{ben} RNA local	P_{del} RNA local	P_{null} RNA piMOM	P_{ben} RNA piMOM	P_{del} RNA piMOM	P_{null} GWAS local	P_{ben} GWAS local	P_{del} GWAS local	P_{null} GWAS piMOM	P_{ben} GWAS piMOM	P_{del} GWAS piMOM	RNA effect local	Dispersion local	RNA effect piMOM	Dispersion piMOM	GWAS effect local	GWAS effect piMOM
98	RANBP2	1.00	0.00	0.00	1.00	0.00	0.00	0.00	0.00	1.00	1.00	0.00	0.00	x	x	x	x	0.27	x
99	RAP1GAP	0.22	0.10	0.68	0.15	0.83	0.02	1.00	0.00	0.00	1.00	0.00	0.00	0.58	1.5	0.6	1.49	x	x
100	RHOQ	1.00	0.00	0.00	1.00	0.00	0.00	0.00	0.00	1.00	1.00	0.00	0.00	x	x	x	x	8.8	x
101	S100B	1.00	0.00	0.00	1.00	0.00	0.00	0.00	0.00	1.00	1.00	0.00	0.00	x	x	x	x	0.47	x
102	SCN1A	1.00	0.00	0.00	1.00	0.00	0.00	0.00	0.00	1.00	1.00	0.00	0.00	x	x	x	x	2.85	x
103	SCRN2	1.00	0.00	0.00	1.00	0.00	0.00	0.00	0.00	1.00	1.00	0.00	0.00	x	x	x	x	2.57	x
104	SEMA3B	1.00	0.00	0.00	1.00	0.00	0.00	0.00	1.00	0.00	1.00	0.00	0.00	x	x	x	x	8.22	x
105	SERINC3	1.00	0.00	0.00	1.00	0.00	0.00	0.00	1.00	0.00	1.00	0.00	0.00	x	x	x	x	0.1	x
106	SLC24A2	1.00	0.00	0.00	1.00	0.00	0.00	1.00	0.00	0.00	0.00	0.00	1.00	x	x	x	x	x	0.67
107	SLC25A29	1.00	0.00	0.00	1.00	0.00	0.00	0.00	0.00	1.00	1.00	0.00	0.00	x	x	x	x	0.03	x
108	SYT13	1.00	0.00	0.00	1.00	0.00	0.00	0.00	1.00	0.00	1.00	0.00	0.00	x	x	x	x	2.34	x
109	SYT5	1.00	0.00	0.00	1.00	0.00	0.00	0.00	1.00	0.00	1.00	0.00	0.00	x	x	x	x	25.12	x
110	TIMM22	1.00	0.00	0.00	1.00	0.00	0.00	0.00	1.00	0.00	1.00	0.00	0.00	x	x	x	x	69.37	x
111	TLR6	1.00	0.00	0.00	1.00	0.00	0.00	0.00	0.00	1.00	1.00	0.00	0.00	x	x	x	x	4.82	x
112	TMEM125	1.00	0.00	0.00	1.00	0.00	0.00	0.00	0.00	1.00	1.00	0.00	0.00	x	x	x	x	0.17	x
113	TMEM181	1.00	0.00	0.00	1.00	0.00	0.00	0.00	0.00	1.00	1.00	0.00	0.00	x	x	x	x	0.17	x
114	TMEM246	1.00	0.00	0.00	1.00	0.00	0.00	0.00	1.00	0.00	1.00	0.00	0.00	x	x	x	x	0.54	x
115	TMTC1	1.00	0.00	0.00	1.00	0.00	0.00	1.00	0.00	0.00	0.00	1.00	0.00	x	x	x	x	x	1.33
116	TPI1	1.00	0.00	0.00	1.00	0.00	0.00	0.00	0.00	1.00	1.00	0.00	0.00	x	x	x	x	15.83	x
117	TRPS1	1.00	0.00	0.00	1.00	0.00	0.00	0.00	0.00	1.00	1.00	0.00	0.00	x	x	x	x	3.56	x
118	TUFM	1.00	0.00	0.00	1.00	0.00	0.00	0.00	0.00	1.00	1.00	0.00	0.00	x	x	x	x	25.96	x
119	UQCR10	1.00	0.00	0.00	1.00	0.00	0.00	0.00	1.00	0.00	1.00	0.00	0.00	x	x	x	x	2.07	x
120	UQCRC1	1.00	0.00	0.00	1.00	0.00	0.00	0.00	0.00	1.00	1.00	0.00	0.00	x	x	x	x	0.39	x
121	WDR12	1.00	0.00	0.00	1.00	0.00	0.00	0.00	0.00	1.00	1.00	0.00	0.00	x	x	x	x	1.77	x
122	XYLT1	1.00	0.00	0.00	1.00	0.00	0.00	1.00	0.00	0.00	0.00	1.00	0.00	x	x	x	x	x	1.36
123	ZDHHC20	1.00	0.00	0.00	1.00	0.00	0.00	0.00	0.00	1.00	1.00	0.00	0.00	x	x	x	x	0.22	x

Table 3: Genes identified as non-null in either the TG RNA only model or GWAS only model with proportion of time spent in each group and effect sizes as in Manuscript Table 1. The top 94 genes in the GWAS only local model are reported (a cutoff of 0.9999) as opposed to all genes in the median model which is used for the other three models.

References

Agarwal, D., Sandor, C., Volpato, V., Caffrey, T.M., Monzón-Sandoval, J., Bowden, R. et al (2020). A single-cell atlas of the human substantia nigra reveals cell-specific pathways associated with neurological disorders. *Nature communications* **11,** 4183–4183.

Albert-Gascó, H., Ros-Bernal, F., Castillo-Gómez, E. and Olucha-Bordonau, F.E. (2020). MAP/ERK signaling in developing cognitive and emotional function and its effect on pathological and neurodegenerative processes. *Int J Mol Sci.* **21,** 4471.

Bagetta, V., Ghiglieri, V., Sgobio, C., Calabresi, P. and Picconi, B. (2010). Synaptic dysfunction in Parkinson's disease. *Biochemical Society Transactions* **38,** 493–497.

Barbieri, M.M. and Berger, J.O. (2004). Optimal predictive model selection. *The Annals of statistics* **32,** 870–897.

Benjamini, Y. and Hochberg, Y. (1995). Controlling the false discovery rate: a practical and powerful approach to multiple testing. *Journal of the Royal Statistical Society Series B-Statistical Methodology* **57,** 289–300.

Bonham, L.W., Karch, C.M., Fan, C.C., Tan, C., Geier, E.G., Wang, Y. et al (2018). Cxcr4 involvement in neurodegenerative diseases. *Translational psychiatry* **8,** 73.

Bose, A. and Beal, M.F. (2019). Mitochondrial dysfunction and oxidative stress in induced pluripotent stem cell models of Parkinson's disease. *Eur J Neurosci.* **49,** 525–532.

Brehm, N., Bez, F., Carlsson, T., Kern, B., Gispert, S., Auburger, G. et al (2015). A genetic mouse model of Parkinson's disease shows involuntary movements and increased postsynaptic sensitivity to apomorphine. *Molecular neurobiology* **52,** 1152–1164.

Chang, M., Zhang, Y., Hui, Z., Wang, D. and Guo, H. (2020). IFRD1 regulates the asthmatic responses of airway via NF-κB pathway. *Molecular Immunology* **127,** 186–192.

Chuang, Y., Lu, A., Paul, K., Folle, A., Bronstein, J., Bordelon, Y. et al (2019). Longitudinal epigenome-wide methylation study of cognitive decline and motor progression in Parkinson's disease. *J Parkinsons Dis.* **9,** 389–400.

Clayton, D.F. and George, J.M. (1998). The synucleins: a family of proteins involved in synaptic function, plasticity, neurodegeneration and disease. *Trends in neurosciences* **21,** 249–254.

Esposito, G., Ana Clara, F. and Verstreken, P. (2012). Synaptic vesicle trafficking and Parkinson's disease. *Developmental neurobiology* **72,** 134–144.

Fisher, R. (1929). Tests of significance in harmonic analysis. *Proceedings of the Royal Society of London Series A-Mathematical and Physical Sciences* **125,** 54–59.

Gelman, A. (2014). *Bayesian data analysis.* CRC Press, Boca Raton, 3rd edition.

Gerard, D. (2020). Data-based RNA-seq simulations by binomial thinning. *BMC bioinformatics* **21,** 206–206.

Gu, X.J., Su, W.M., Dou, M., Jiang, Z., Duan, Q.Q., Yin, K.F. et al (2023). Expanding causal genes for Parkinson's disease via multi-omics analysis. *npj Parkinson's Disease* **9,**.

Hu, W., Wang, M., Sun, G., Zhang, L. and Lu, H. (2024). Early b cell factor 3 (EBF3) attenuates Parkinson's disease through directly regulating contactin-associated protein-like 4 (CNTNAP4) transcription: An experimental study. *Cellular Signalling* **,,** 111–139.

Kochmanski, J., Kuhn, N.C. and Bernstein, A.I. (2022). Parkinson's disease-associated, sex-specific changes in DNA methylation at PARK7 (DJ-1), SLC17A6 (VGLUT2), PTPRN2 (IA-2β), and NR4A2 (NURR1) in cortical neurons. *npj Parkinson's Disease* **8,** 120.

Lev, N., Melamed, E. and Offen, D. (2003). Apoptosis and Parkinson's disease. *Progress in Neuro-Psychopharmacology and Biological Psychiatry* **27,** 245–250.

Li, L., Wang, H., Li, H., Lu, X., Gao, Y. and Guo, X. (2022). Long noncoding rna bace1-antisense transcript plays a critical role in Parkinson's disease via microRNA-214-3p/cell death-inducing p53-target protein 1 axis. *Bioengineered* **13,** 10889–10901.

Liu, H., Ho, P.W.L., Leung, C.T., Pang, S.Y.Y., Chang, E.E.S., Choi, Z.Y.K. et al (2021). Aberrant mitochondrial morphology and function associated with impaired mitophagy and DNM1L-MAPK/ERK signaling are found in aged mutant Parkinsonian LRRK2R1441G mice. *Autophagy* **17,** 3196–3220.

Liu, Y. and Xie, J. (2020). Cauchy combination test: a powerful test with analytic p-value calculation under arbitrary dependency structures. *Journal of the American Statistical Association* **115,** 393–402.

Liu, Y.X., Wang, J., Guo, J., Wu, J., Lieberman, H.B. and Yin, Y. (2008). DUSP1 is controlled by p53 during the cellular response to oxidative stress. *Molecular Cancer Research* **6,** 624–633.

Ma, J., Dong, L., Chang, Q., Chen, S., Zheng, J., Li, D. et al (2023). CXCR4 knockout induces neuropathological changes in the MPTP-lesioned model of Parkinson's disease. *Biochimica et Biophysica Acta (BBA)-Molecular Basis of Disease* **1869,** 166597.

Mochizuki, H., Goto, K., Mori, H. and Mizuno, Y. (1996). Histochemical detection of apoptosis in Parkinson's disease. *Journal of the neurological sciences* **137,** 120–123.

Montgomery, S.B., Dermitzakis, E.T., Sammeth, M., Gutierrez-Arcelus, M., Lach, R.P., Ingle, C. et al (2010). Transcriptome genetics using second generation sequencing in a caucasian population. *Nature (London)* **464,** 773–777.

Moon, H.E. and Paek, S.H. (2015). Mitochondrial dysfunction in Parkinson's disease. *Experimental neurobiology* **24,** 103.

Morais, V.A., Verstreken, P., Roethig, A., Smet, J., Snellinx, A., Vanbrabant, M. et al (2009). Parkinson's disease mutations in PINK1 result in decreased complex I activity and deficient synaptic function. *EMBO molecular medicine* **1,** 99–111.

Nalls, M.A., Pankratz, N., Lill, C.M., Do, C.B., Hernandez, D.G., Saad, M. et al (2014). Large-scale meta-analysis of genome-wide association data identifies six new risk loci for Parkinson's disease. *Nature genetics* **46,** 989–+.

Perrett, R.M., Alexopoulou, Z. and Tofaris, G.K. (2015). The endosomal pathway in Parkinson's disease. *Molecular and Cellular Neuroscience* **66,** 21–28.

Pickrell, J.K., Gilad, Y., Pritchard, J.K., Marioni, J.C., Pai, A.A., Degner, J.F. et al (2010). Understanding mechanisms underlying human gene expression variation with rna sequencing. *Nature (London)* **464,** 768–772.

Schilder, B.M. and Raj, T. (2022). Fine-mapping of Parkinson's disease susceptibility loci identifies putative causal variants. *Human Molecular Genetics* **31,** 888–900.

Scott, J.G. and Berger, J.O. (2010). Bayes and empirical-Bayes multiplicity adjustment in the variable-selection

problem. *The Annals of Statistics.* **38,** 2587–2619.

Singh, P.K. and Muqit, M.M. (2020). Parkinson's: a disease of aberrant vesicle trafficking. *Annual review of cell and developmental biology* **36,** 237–264.

Strobbe, D., Robinson, A.A., Harvey, K., Rossi, L., Ferraina, C., De Biase, V. et al (2018). Distinct mechanisms of pathogenic DJ-1 mutations in mitochondrial quality control. *Frontiers in Molecular Neuroscience* **11,** 68.

Tan, E.K., Chao, Y.X., West, A., Chan, L.L., Poewe, W. and Jankovic, J. (2020). Parkinson disease and the immune system—associations, mechanisms and therapeutics. *Nature Reviews Neurology* **16,** 303–318.

Tatton, W.G., Chalmers-Redman, R., Brown, D. and Tatton, N. (2003). Apoptosis in Parkinson's disease: signals for neuronal degradation. *Ann Neurol.* **53,** S61–S72.

Toskas, K., Yaghmaeian-Salmani, B., Skiteva, O., Paslawski, W., Gillberg, L., Skara, V. et al (2022). PRC2-mediated repression is essential to maintain identity and function of differentiated dopaminergic and serotonergic neurons. *Science Advances* **8,** eabo1543.

Usenko, T., Bezrukova, A., Basharova, K., Panteleeva, A., Nikolaev, M., Kopytova, A. et al (2021). Comparative transcriptome analysis in monocyte-derived macrophages of asymptomatic gba mutation carriers and patients with GBA-associated Parkinson's disease. *Genes* **12,** 1545.

Wang, J., Zhou, J.Y., Kho, D., Reiners Jr, J.J. and Wu, G.S. (2016). Role for DUSP1 (dual-specificity protein phosphatase 1) in the regulation of autophagy. *Autophagy* **12,** 1791–1803.

Zaltieri, M., Longhena, F., Pizzi, M., Missale, C., Spano, P., Bellucci, A. et al (2015). Mitochondrial dysfunction α-Synuclein synaptic pathology in Parkinson's disease: who's on first? *Parkinson's disease* **2015,**.

Zhang, W., Zhou, M., Lu, W., Gong, J., Gao, F., Li, Y. et al (2020). CNTNAP4 deficiency in dopaminergic neurons initiates Parkinsonian phenotypes. *Theranostics* **10,** 3000.

Zhao, C., Datta, S., Mandal, P., Xu, S. and Hamilton, T. (2010). Stress-sensitive regulation of IFRD1 mRNA decay is mediated by an upstream open reading frame. *Journal of Biological Chemistry* **285,** 8552–8562.
